# Modulation of UPF1 catalytic activity upon interaction of SARS-CoV-2 Nucleocapsid protein with factors involved in nonsense mediated-mRNA decay

**DOI:** 10.1093/nar/gkae829

**Published:** 2024-10-03

**Authors:** Megha Mallick, Volker Boehm, Guangpu Xue, Mark Blackstone, Niels H Gehring, Sutapa Chakrabarti

**Affiliations:** Institute of Chemistry and Biochemistry, Freie Universität Berlin, Takustr. 6, D-14195 Berlin, Germany; Institute for Genetics, University of Cologne, Cologne 50674, Germany; Center for Molecular Medicine Cologne (CMMC), University of Cologne, Cologne 50931, Germany; Institute of Chemistry and Biochemistry, Freie Universität Berlin, Takustr. 6, D-14195 Berlin, Germany; Institute of Chemistry and Biochemistry, Freie Universität Berlin, Takustr. 6, D-14195 Berlin, Germany; Institute for Genetics, University of Cologne, Cologne 50674, Germany; Center for Molecular Medicine Cologne (CMMC), University of Cologne, Cologne 50931, Germany; Institute of Chemistry and Biochemistry, Freie Universität Berlin, Takustr. 6, D-14195 Berlin, Germany

## Abstract

The RNA genome of the SARS-CoV-2 virus encodes for four structural proteins, 16 non-structural proteins and nine putative accessory factors. A high throughput analysis of interactions between human and SARS-CoV-2 proteins identified multiple interactions of the structural Nucleocapsid (N) protein with RNA processing factors. The N-protein, which is responsible for packaging of the viral genomic RNA was found to interact with two RNA helicases, UPF1 and MOV10 that are involved in nonsense-mediated mRNA decay (NMD). Using a combination of biochemical and biophysical methods, we investigated the interaction of the SARS-CoV-2 N-protein with NMD factors at a molecular level. Our studies led us to identify the core NMD factor, UPF2, as an interactor of N. The viral N-protein engages UPF2 in multipartite interactions and can negate the stimulatory effect of UPF2 on UPF1 catalytic activity. N also inhibits UPF1 ATPase and unwinding activities by competing in binding to the RNA substrate. We further investigate the functional implications of inhibition of UPF1 catalytic activity by N in mammalian cells. The interplay of SARS-CoV-2 N with human UPF1 and UPF2 does not affect decay of host cell NMD targets but might play a role in stabilizing the viral RNA genome.

## Graphical Abstract

**Graphical Abstract:**
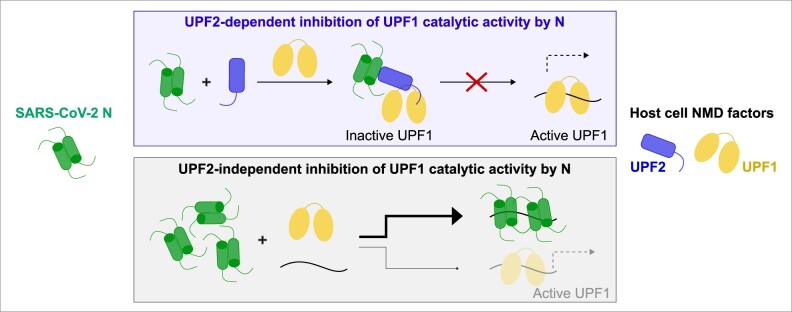

## Introduction

Coronaviruses belong to the order *Nidovirales*, which derives its name from the ‘nested’-subgenomic (sg) mRNA (similar 5′-ends and identical 3′-ends) transcribed upon infection of host cells (1,2). Approximately two-thirds of the 5′-end of the RNA genome of severe acute respiratory syndrome-related (SARS) coronaviruses comprise open reading frames (ORFs) 1a and 1b that are related by a 1 nucleotide ribosomal frameshift. They encode for two large polyproteins that are proteolytically processed to yield 16 non-structural proteins (Nsps) (3,4). These Nsps together form the replication-transcription complex (RTC) which is necessary for viral RNA synthesis (5,6). Discontinuous transcription of the 3′-end of the RNA genome leads to production of negative-sense sgRNAs that serve as templates for nested positive-strand sg-mRNAs, which can be translated to produce 4 structural proteins (Spike, Envelope, Membrane and Nucleocapsid, also referred to as S, E, M and N, respectively) and multiple accessory proteins (7,8). Continuous transcription from the 3′-end leads to synthesis of a full-length negative-sense RNA, which is used as a template for generation of new positive-sense genomic RNA. The viral genomic RNA is packaged by the Nucleocapsid (N) protein and enveloped by the remaining structural proteins to produce a helical nucleocapsid, in contrast to icosahedral nucleocapsids common to other positive-strand RNA viruses (9,10).

The sg-mRNAs resemble host cell mRNAs in that they are capped at the 5′-end and polyadenylated at the 3′-end, enabling recognition of these mRNAs by the host cell translation machinery (11). Although the 5′-leader sequence is identical in all sg-mRNAs, the lengths of the 3′-untranslated regions (3′-UTRs) greatly differ, ranging from 100 to 4000 nucleotides, depending on the site of initiation of discontinuous transcription. The genomic RNA, the 5′-end of which serves as a template for translation of the two large polyproteins, has the longest 3′-UTR of ∼10 kb (12). In eukaryotes, transcripts with long 3′-UTRs are known to potentially trigger the nonsense-mediated mRNA decay (NMD) pathway (13–15). The NMD pathway is a translation-dependent mechanism to detect and degrade mRNAs with unusual features such as premature termination codons (PTCs) or long 3′-UTRs (16,17). Certain sg-mRNAs as well as the genomic RNA of coronaviruses are, therefore, putative NMD targets. Interestingly, a study by Wada and co-workers using the murine hepatitis virus (MHV) as a model coronavirus reported an intricate interplay of the MHV N-protein with the NMD pathway at different stages of infection (18). At an early stage of infection, when levels of N are low, viral replication was shown to be impeded by NMD. Upon sufficient accumulation of N in the later stages of infection, NMD is successfully inhibited by the N-protein, allowing for efficient viral replication. A recent study by Gordon and co-workers on mapping the SARS-CoV-2 – human interactome revealed interactions between the SARS-CoV-2 N-protein (hereafter referred to as N) and the RNA helicases, UPF1 and MOV10, which are known to play important roles in the NMD pathway (19).

The RNA helicase UPF1 (***Up***-***f***rameshift protein *1*) is a central component of the NMD pathway and along with the core NMD factors UPF2 and UPF3, is conserved from yeast to humans (20–22). While the role of UPF1 in NMD was primarily thought to be to remodel nonsense mRNPs prior to degradation, it now appears that the RNA-dependent ATPase activity of UPF1 is also essential for discerning between cognate and non-cognate NMD substrates (23,24). Furthermore, UPF1 also serves as a phosphorylation-dependent protein–protein interaction platform to recruit factors that are important for progression of NMD and ultimately, degradation of the target mRNA (25–28). The low basal catalytic activity of UPF1 is greatly stimulated upon binding to UPF2, which induces a large conformational change that switches the helicase from an inactive RNA-clamping mode to an active RNA-unwinding mode (29,30). Recently, it was shown that UPF2 triggers release of UPF1 from RNA, suggesting a rapid activation-and-dissociation mode of action of the helicase and its activator (31). Although UPF1 is a key player of the NMD pathway, it is not the only RNA helicase involved. The helicases DHX34 and MOV10 were also shown to be involved in the NMD pathway, albeit at very different stages. DHX34 is thought to function as a scaffold to recruit UPF1 to the PI3K-like kinase, SMG1, to mediate its phosphorylation while MOV10 acts at a later stage of NMD, presumably to unwind RNA secondary structures in the 3′-UTR and facilitate decay (32,33). The observed interactions of N with UPF1 and MOV10, as determined by affinity-purification mass-spectrometry (AP-MS), suggest a direct perturbation of the NMD pathway by the N-protein and corroborates previous observations on the impact of MHV N on NMD (19). However, AP-MS does not distinguish between direct protein–protein interactions and those bridged by additional factors or adaptor proteins. Therefore, we adopted a biochemical approach to systematically test the interaction of SARS-CoV-2 N with the NMD factors, UPF1, UPF2 and MOV10, to elucidate the molecular mechanisms of the interplay of coronavirus N-proteins and NMD. Surprisingly, we found that the N-protein has no significant direct binding to UPF1 or MOV10 but instead interacts with UPF2. Using further biochemical and biophysical assays, we showed that N engages UPF2 in multi-partite interactions and indirectly associates with UPF1 to repress its catalytic activity. However, inhibition of UPF1 activity does not translate to inhibition of host cell NMD by N. We speculate that the N-protein might specifically inhibit NMD of viral genomic and sub-genomic mRNA while leaving host-cell NMD targets unaffected.

## Materials and methods

### Protein expression and purification

All human UPF1 (except for full-length UPF1) and UPF2 constructs, MOV10_fus_ as well as SARS-CoV-2 N variants used in this study were expressed in *Escherichia coli* as 6X-His or His-GST fusions that are cleavable by TEV or HRV-3C protease. Details of the different constructs used in this study are provided in Supplementary Table S1. The primers used to generate the different N expression constructs are described in Supplementary Table S2. The SARS-CoV-2 Nfl plasmid was a gift from Markus Wahl. All plasmids produced for this study were verified by Sanger sequencing. Protein expression in *E. coli* BL21(DE3) star pRare cells was induced by addition of *iso*-propyl β-d-1-thiogalactopyranoside (IPTG) and performed at 18°C for 20 h. Full-length UPF1 was expressed using a baculovirus expression system (see Supplementary methods).

All proteins were first subjected to Nickel-affinity chromatography, followed by Heparin-affinity chromatography and size-exclusion. Briefly, cells expressing the desired protein were resuspended in lysis buffer A (50 mM Tris–HCl pH 7.5, 500 mM NaCl, 10 mM imidazole and 10% glycerol) supplemented with 1 mM phenylmethylsulphonyl fluoride (PMSF) and 0.5 mg of DNase I. All buffers used for purification of UPF1 and MOV10_fus_ were further supplemented with 1 mM MgCl_2_, 1 μM ZnCl_2_ and 0.1 M urea. The cell suspension was lysed by sonication, following which the lysate containing soluble protein was separated from the cell debris by centrifugation and filtration. The clarified cell lysate was incubated with Ni^2+^-affinity resin (Machery-Nagel #745400.100). His-tagged protein was eluted in elution buffer B (20 mM Tris-HCl pH 7.5, 150 mM NaCl, 300 mM imidazole and 10% glycerol). The one-step purified protein was then loaded onto a HiTrap Heparin Sepharose HP column (GE Healthcare) and eluted from it in a salt gradient of 100–1000 mM NaCl. Size exclusion chromatography (SEC) using Superdex 75 or Superdex 200 columns (GE Healthcare) was performed in SEC buffer C (20 mM Tris–HCl pH 7.5, 150 mM NaCl, 5% glycerol and 2 mM DTT) as a final step to ensure homogeneity of the isolated protein. For all N-proteins, the pH of the SEC buffer was maintained at 8.0. In every case, the purity of the target protein was verified by SDS-PAGE after each chromatographic step.

To purify the UPF1–UPF2 and UPF2–UPF3 complexes, proteins were mixed in a 1 to 1.2 molar ratio (with UPF1 in excess for the UPF1–UPF2 complex, and UPF3 in excess for the UPF2–UPF3 complex) for 16 h at 4°C and resolved on a Superdex 200 column using SEC buffer C.

### GST pull-down assays

8 μg of bait and prey proteins were mixed and diluted to 40 μl in GST pulldown buffer (20 mM HEPES pH 7.5, 150 mM NaCl, 10% glycerol and 0.1% NP-40). 0.4 μg of the protein mixture was used as an input for each reaction. The protein mixtures were incubated on ice for 1 h, following which 12 μl of a 50% Glutathione Sepharose resin (GE Healthcare) was added to each sample. The mixture was further supplemented with 150 μl of GST-pulldown buffer and incubated at 4°C for 90 min. The beads were extensively washed with the same buffer before eluting the bound proteins in GST-pulldown buffer supplemented with 20 mM glutathione. The inputs and elutions were analysed by SDS-PAGE and Coomassie staining.

### Isothermal titration calorimetry (ITC)

ITC analyses were conducted to determine the binding affinities of UPF2 constructs (UPF2_S_, UPF2-MIF4G3 and UPF2-U1BD) and the N_IDR1-core_ protein at 10°C on an iTC200 instrument (MicroCal). All proteins were purified by SEC (Superdex 200 10/300) using a SEC/ITC buffer (20mM Tris–HCl, pH 8.0, 150 mM NaCl and 5% glycerol) immediately before their use in ITC. 40 μl of 200 μM of each UPF2 protein was loaded in the syringe and titrated against 250 μl of 20 μM N_IDR1-core_ in a series of injections. Twenty injections were performed in total, where the first injection was of 0.5 μl, followed by 19 injections of 2 μl each, at 180-s intervals. Analysis of N_core_ binding to UPF2_S_ was carried out using an identical procedure. The data were analysed with the MicroCal PEAQ-ITC Analysis Software (Malvern Instruments) by subtracting baseline and offset. The N variants were considered as dimers for concentration measurements and data analyses in all experiments requiring molar concentrations of proteins.

### Analytical size exclusion (SEC)

1 nmol of the UPF1–UPF2 and UPF2–UPF3 complexes were mixed with 1 nmol of N-proteins to a final volume of 50 μl in SEC buffer D (20 mM HEPES pH 7.5, 150 mM NaCl, 2% glycerol, 1 mM MgCl_2_, 1 μM ZnCl_2_, 2 mM DTT). Similarly, for a 1:1 and 1:2 UPF1–RNA–N complex, 0.5 nmol of UPF1, 0.5 nmol of U_45_ RNA and 0.5 nmol (for 1:1 mixture) or 1 nmol (for 1:2 mixture) of N_ΔL_ were mixed and incubated at room temperature for 1 h. A mixture of 0.5 nmol of UPF1 and 0.5 nmol of N_ΔL_ was also analysed to assess complex formation in the absence of RNA. The protein or protein-RNA mixtures were resolved on a Superdex 200 Increase 3.2/300 column in SEC buffer D. The peak fractions were analysed by SDS-PAGE and Coomassie staining to visualize proteins. Quantification of protein levels in SDS gels was done by performing a densitometric analysis of the bands using ImageJ. To detect RNA, 3 μl of each peak fraction was 5′-end labelled with [γ-^32^P]-ATP using T4 polynucleotide kinase. The labelling reactions were resolved on a 12% urea–PAGE gel and visualized by phosphorimaging.

### Fluorescence-based unwinding assay

The sequences of the RNA and DNA strands of the RNA:DNA hybrid and that of the trap DNA strand are provided in Supplementary methods. The DNA strand of the RNA:DNA hybrid was labelled at the 5′-end with Alexa Fluor 488 and the complementary trap DNA strand was labelled at the 3′-end with a quencher (Blackhole quencher 1).

The RNA substrate used in this helicase assay was prepared by *in vitro* transcription (IVT) from a linearized double-stranded (ds) DNA template. The DNA oligos used as the template for IVT are described in Supplementary Table S2. The RNA:DNA duplex substrate for the unwinding assay was freshly prepared prior to every set of experiments by incubating the DNA and RNA strands in a 7:11 ratio with 2 mM magnesium acetate and 1× unwinding buffer (10 mM MES pH 6.5, 50 mM potassium acetate, 0.1 mM EDTA) at 95°C for 210 s, followed by slow cooling of the mixture down to 30°C. For each replicate, an initial reaction mixture was prepared which included 75 nM of freshly assembled RNA-DNA duplex in 1x unwinding buffer, 2 mM magnesium acetate, 2 mM freshly prepared DTT, and 300 nM UPF1, 600 nM UPF2 wherever mentioned, and 300–1200 nM of Nfl. For reactions comparing the effects of N_L-DD_ with Nfl, only 75 and 300 nM of N-proteins were used. The reaction mixture containing UPF1 or UPF1–UPF2 was incubated at 25°C for 10 min prior to adding N. The mixture was incubated for another 10 min after addition of N. Trap DNA was added at the very end to a final concentration of 0.56 μM. The reaction mixture was prepared in the dark and transferred to a 384-well plate (PerkinElmer OptiPlate 384-F). 2 mM ATP was injected into each well using the injector module of the Spark multimode microplate reader (Tecan Life Sciences). The fluorescence was monitored for 30 min at 30°C on the same instrument. The measured fluorescence intensities were normalized to the 0-time point (baseline) for each condition to obtain relative fluorescence. A more detailed protocol in the form of a flow chart can be found in Supplementary methods.

### ATPase assay

The ATPase activity of UPF1 alone and in presence of its activator UPF2, and different amounts of Nfl was determined by quantifying the amount of inorganic phosphate released upon ATP hydrolysis using a coupled colorimetric assay (EnzCheck Phosphate Kit, Thermo Fischer Scientific). The protein mixtures were pre-incubated in a reaction volume of 150 μl with 1 μg poly-U-RNA, 40 nmol MESG (2-amino-6-mercapto-7-methylpurine ribonucleoside) and 0.5 U purine-nucleoside phosphorylase in an ATPase reaction buffer (50 mM MES pH 6.5, 50 mM potassium acetate, 5 mM magnesium acetate and 2 mM DTT) at 30°C for 20 min. The reaction was initiated by addition of 1 mM ATP (final concentration in a final volume of 200 μl) in a 96-well plate (Greiner). 2-amino-6-mercapto-7-methylpurine produced upon cleavage of MESG by the inorganic phosphate generated from the ATPase reaction was detected by measuring absorbance at 360 nm on a Spark multimode microplate reader (Tecan Life Sciences). The reaction was monitored over a period of 20 min at 60-s intervals. The amount of UPF1 was kept constant (i.e. 6 pmol or final concentration of 200 nM) in each experiment, and UPF2 was added in 2-fold molar excess of UPF1 (12 pmol or final concentration of 400 nM). Nfl was added as indicated in the figures. The end point of the ATPase reaction of the UPF1-UPF2_S_ mixture in each case was set to 1 or 100%, and all other values were normalized to this maximum value.

### Cell culture

Flp-In T-REx-293 (human, female, embryonic kidney, epithelial; Thermo Fisher Scientific, RRID:CVCL_U427) cells were cultured in high-glucose, GlutaMAX DMEM (Gibco) supplemented with 9% fetal bovine serum (Gibco) and 1× penicillin streptomycin (Gibco). The cells were cultivated at 37°C and 5% CO_2_ in a humidified incubator. Stable cell lines were generated by using the PiggyBac (PB) Transposon system with the cumate-inducible PB-CuO-MCS-BGH-EF1-CymR-Puro vector. This vector was modified from the original vector (PB-CuO-MCS-IRES-GFP-EF1α-CymR-Puro (System Biosciences)) by replacing the IRES-GFP cassette with a BGH polyA signal. Inserts coding for N proteins were cloned from SARS-CoV-2 Nfl plasmid (mentioned above). For cumate-inducible NMD reporter plasmids, the 75-nt long 5′ leader sequence (5′-TRS-L) of CoV-2 was cloned directly upstream of beta-globin reporter genes (WT or PTC39 variant).

The sequence of TRS-L is:

5′-attaaaggtttataccttcccaggtaacaaaccaaccaactttcgatctcttgtagatctgttctctaaacgaac-3′.

Co-transfection of cells with protein-encoding and NMD reporter-encoding plasmids was performed using a calcium phosphate-based system with BES buffered saline (BBS). Stably integrated cells were selected using media containing 1.5 μg/ml puromycin (InvivoGen) for a week. To express the reporters and N-terminally FLAG-tagged protein constructs, 5.0 × 10^5^ cells were seeded in 6-well plates with 30 μg/ml cumate (4-isopropylbenzoic acid) added directly. Fresh medium with cumate and 0, 0.1 or 1 μM SMG1 kinase inhibitor (SMG1i) was added the next day, and the cells were harvested after another 24 h.

### Western blotting

SDS-polyacrylamide gel electrophoresis and immunoblot analysis were performed using protein samples harvested with RIPA buffer (50 mM Tris/HCl pH 8.0, 0.1% SDS, 150 mM NaCl, 1% IGEPAL, 0.5% deoxycholate). For protein quantification, the Pierce Detergent Compatible Bradford Assay Reagent (Thermo Fisher Scientific) was used. Monoclonal mouse anti-FLAG primary antibody (F3165, 1:3000 dilution, Sigma Aldrich), monoclonal mouse anti-alpha-Tubulin primary antibody (T6074, 1:10000 dilution, Sigma Aldrich) and multi-rAb HRP-goat anti-mouse recombinant secondary antibody (RGAM001, 1:10000 dilution, Proteintech) were used to detect FLAG-tagged proteins and Tubulin as loading control. Detection was performed with ECL Prime Western Blotting Detection Reagent (Amersham) and the Vilber Fusion FX6 Edge imaging system (Vilber Lourmat).

### RNA extraction

For RNA extraction, the cells were harvested with 1 ml in-house prepared TRI reagent per 6 well (34) and RNA was isolated according to standard protocols. 150 μl 1-bromo-3-chloropropane (Sigma-Aldrich) was used to induce phase separation. The washed RNA pellet was dissolved in 20 μl RNase-free water by incubating for 10 min on a shaking 65°C heat block.

### Quantitative reverse transcriptase (RT)-PCR

Reverse transcription was performed with 4 μg of total RNA in a 20 μl reaction volume with 10 μM VNN-(dT)20 primer and the GoScript Reverse Transcriptase (Promega). Probe-based multiplex quantitative RT-PCRs were performed with the PrimeTime Gene Expression Master Mix (IDT), 2% of cDNA per reaction, and the CFX96 Touch Real-Time PCR Detection System (Bio-Rad). PrimeTime qPCR Assays containing primers and probes were purchased from IDT (B2M = Hs.PT.58v.18759587, ZFAS1 = Hs.PT.58.25163607, GAS5 = Hs.PT.58.24767969) and used at 1× final concentration according to the manufacturer's instruction. Each biological replicate was repeated in technical triplicates and the average Ct (threshold cycle) value was measured. The housekeeping gene B2M (FAM-labelled) Ct values were subtracted from the target (ZFAS1, Cy5-labelled or GAS5, SUN-labelled) values to receive the ΔCt. To calculate the mean log2 fold changes, three biologically independent experiments were used. The log_2_ fold changes are visualized as single data points and mean. Quantitative RT-PCR for globin reporter was performed with the GoTaq qPCR Master Mix (Promega) using 2% of cDNA in 10 μl reactions, 0.2 μM final concentration of sense (AAGGCTCATGGCAAGAAAG) and antisense (ACACCAGCCACCACTTTC) primer. Ct values were normalized to B2M from the probe-based experiment. Statistical analysis was performed using the tukey_hsd (Tukey Honest Significant Differences) function from the rstatix R package. End-point PCR for SRSF2 was performed using MyTaq Red Mix (Bioline), 2% of cDNA (template) and 0.2 μM final concentration of sense (GAATCCAAATCCAGGTCGC) and antisense (CCAGTTGCTTGTTCCAAGGA) primer. After 30 PCR cycles, the PCR products were resolved by electrophoresis on ethidium bromide-stained, 1% agarose TBE gels and detected by trans-UV illumination using the Gel Doc XR+ (Bio-Rad) and Image Lab software (version 5.1).

### RNA-seq

For RNA-Seq, human embryonic kidney 293 (HEK293) cells were cultured in DMEM high glucose media supplemented with 10% FBS and 1% penicillin/streptomycin (Invitrogen). The cells were seeded in 6-well plates and grown to 75% confluence in DMEM high glucose media supplemented only with FBS. 1 μg plasmid of pCDNA3.1-HA-SARS-CoV-2 N were transfected using lipofectamine 2000 (Invitrogen). Cells were harvested for RNA extraction 48 h post-transfection. HEK293 cells transfected with an empty vector served as a negative control. Total RNA was extracted from cells using RNATri (Bio&Sell), followed by DNase I digestion to remove contaminating DNA. Final RNA concentration was adjusted to 250 ng/μl using RNase-free water. Three independent samples were prepared for each condition. Libraries were prepared using the mRNA enrichment method at BGI Genomics and sequenced using Eukaryotic Strand-specific Transcriptome Resequencing PE150 technique. This yielded around 40 million paired-end 150 nt reads for all samples.

### Computational analyses of RNA-sequencing data

In addition to the dataset generated in this study, five publicly available RNA-sequencing datasets were obtained and analyzed: (i) SMG7-KO + SMG5/SMG6-KD in HEK293 cells (E-MTAB-9330, ArrayExpress database at EMBL-EBI, (35)), (ii) GFP-CoV-2-N overexpression in HEK293 cells (GSE171010, Gene Expression Omnibus (GEO), (36)), (iii) CoV-2-N-2xStrep overexpression in HEK293 cells (PRJEB44716, European Nucleotide Archive, (37)), (iv) CoV-2-N-2xStrep overexpression in Calu-3 cells (PRJEB45515, European Nucleotide Archive, (37)) and (v) SARS-CoV-2 infection in Calu-3 cells (GSE148729, Gene Expression Omnibus (GEO), (38)). Transcript abundance estimates were computed with Salmon (version 1.9.0) (39) with a decoy-aware transcriptome based on GRCh38 GENCODE release 42 transcript annotations (40). After the import of transcript abundances in R (https://www.R-project.org/ version 4.3.0) using tximport (41) (version 1.28), differential gene expression analysis was performed with the DESeq2 R package (42) (version 1.40.1). Genes with less than 10 counts in half the analyzed samples were pre-filtered and discarded. The DESeq2 LFC estimates were shrunk using the apeglm R package (43) (version 1.22.1). Differential transcript expression analysis was performed using the Swish method from the fishpond R package (44) (version 2.6.2) based on 30 inferential replicate datasets drawn by Salmon using Gibbs sampling and imported via tximeta (45). Transcripts were pre-filtered using 10 counts per transcript in at least one condition as cut-off. Significance cut-offs were log_2_FoldChange > 1 & *P*_adj_ < 0.0001 for DESeq2 DGE and log_2_FC > 1 & *q*-value < 0.01 for Swish DTE. All plots were generated using ggplot2 (46) (version 3.4.2), ComplexHeatmap (version 2.18.0) (47) or nVennR (48) (version 0.2.3). The percentage of CoV-2-N reads were quantified using BBDuk from the BBTools framework (https://jgi.doe.gov/data-and-tools/software-tools/bbtools/).

## Results

### SARS-CoV-2 N directly interacts with the NMD factor UPF2

**Figure 1.**
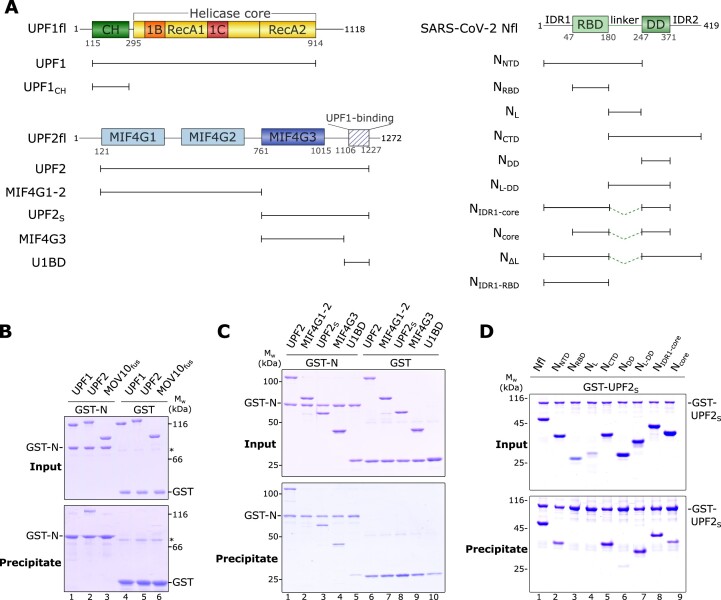
The SARS-CoV-2 Nucleocapsid (N) protein directly interacts with the core NMD factor UPF2. (**A**) Schematic representation of the domain organization of human UPF1, UPF2 and SARS-CoV-2 N, and the constructs used in this study. Structured domains are shown as filled rectangles while intrinsically disordered regions (IDRs) are indicated by lines. (**B**) GST-pulldown assay of UPF1, UPF2 and MOV10_fus_ (a fusion of the N-terminus of MOV10 with the helicase core of UPF1) with GST-N as a bait. GST was used as a negative control in all such assays. The asterisk (*) indicates a contaminant. The top and bottom panels depict the inputs and precipitates, respectively, in this and all other GST-pulldown experiments. GST-N binds UPF2 but not UPF1 or MOV10_fus_. (**C**) GST-pulldown assay to determine the domain of UPF2 that interacts with N. The MIF43G domain is the primary binding site for N, with additional weak interactions mediated by the UPF1-binding domain, U1BD. (**D**) GST-pulldown assay to identify the UPF2-binding region of N. Two IDRs (IDR1 and the inter-domain linker) and the dimerization domain (DD) of N make up a composite binding site for UPF2. No single site on N can mediate a strong interaction with UPF2 but a combination of any two binding sites restores binding comparable to that of full-length N (see also Supplementary Figure S1C). A complete gel including negative controls with GST is shown in Supplementary Figure S1D.

To recapitulate the protein-protein interactions identified in the AP-MS study described above *in vitro*, we performed GST-pulldown assays using full-length GST-tagged N (GST-N) as a bait and UPF1, UPF2 and MOV10 as preys. For this study, we used a variant of UPF1 comprising the cysteine-histidine rich (CH) domain and the helicase core, and lacking the N- and C-terminal unstructured regions. Since full-length UPF2 is proteolytically unstable, we used a truncated construct lacking 120 and 45 residues at the N- and C-termini, respectively and refer to this as UPF2 (Figure 1A). Full-length MOV10 could not be adequately expressed and purified from bacterial or insect cell expression systems. Therefore, a fusion (MOV10_fus_) of the unique MOV10 N-terminus (residues 1–264) with the highly homologous helicase core of UPF1 (residues 295–914) was employed instead. GST-N does not interact with UPF1 or MOV10_fus_*in vitro* (Figure 1B). A full-length UPF1 protein (UPF1fl) expressed and purified using a baculovirus system also does not bind N (Supplementary Figure S1A). However, a weak interaction between the proteins was observed upon addition of poly-uridyl RNA (poly-U) to the mixture of GST-N and UPF1, and upon using GST-UPF1 as a bait for the pulldown (Supplementary Figure S1B). Taken together, our results suggest that there is, at best, a weak direct interaction between the N-protein and UPF1.

In contrast to UPF1 and MOV10_fus_, UPF2 showed a robust interaction with GST-N (Figure 1B). UPF2 is a multidomain protein with three ***m***iddle of e***IF4G*** (MIF4G) domains, followed by a stretch of low-complexity sequence that is natively unstructured (Figure 1A) (49). This unstructured C-terminal region binds UPF1 and is therefore referred to as the UPF1-binding domain (U1BD) (30,50). Generally, MIF4G domains are well-known protein–protein interaction platforms. Although no binding partners for the MIF4G1 and MIF4G2 domains of UPF2 have been identified till date, the third MIF4G domain interacts with various RNA-processing factors such as the NMD factors UPF3 and SMG1, the double-stranded RNA binding protein STAU1 and the eukaryotic release factor ERF3 (49,51–54). In order to identify the binding site for N on UPF2, we used a series of UPF2 constructs, systematically encompassing or lacking specific domains (Figure 1A), and tested the interaction of these truncated proteins with full-length N by GST-pulldown assays. GST-N showed robust binding to all UPF2 variants that contain the MIF4G3 domain (UPF2, UPF2_S_ and UPF2-MIF4G3) but showed no binding to the MIF4G1-2 domains alone (Figure 1C, lanes 1–4). GST-N also bound UPF2-U1BD, albeit weaker than to variants including the MIF4G3 domain (Figure 1C, compare lane 5 with lanes 3 and 4).

We next proceeded to map the UPF2-binding site on N using a similar approach as that described above. The N-protein has two structured domains: an RNA-binding domain (RBD) and a dimerization domain (DD) that are connected by a linker of low complexity sequence. The RBD and DD are also flanked at the N- and C-terminus, respectively, by intrinsically disordered regions (IDRs) (Figure 1A). We generated a series of truncation constructs of N that either comprise an individual domain or a domain together with its flanking IDRs (Figure 1A), and tested the interaction of these variants with GST-UPF2_S_ in a GST-pulldown assay (Figure 1D). As expected, full-length N (Nfl) showed strong binding to UPF2. Variants of N comprising the structured domains alone (RBD and DD) displayed no appreciable binding to UPF2; while no interaction was observed with the RBD, the DD showed a weak binding to UPF2 (Figure 1D, lanes 3 and 6). However, addition of the flexible linker and the flanking IDR to the structured domains (IDR1 in case of N_RBD_ and IDR2 for N_DD_) restored the interaction with UPF2 in the resultant proteins (N_NTD_ and N_CTD_, Figure 1D, lanes 2 and 5). Although neither the linker nor the DD of N show strong binding to UPF2 on their own, a variant comprising both segments (N_L-DD_) can mediate a robust interaction (Figure 1D, compare lanes 4, 6 and 7). As the extent of binding of the N_CTD_ and N_L-DD_ variants to UPF2 are very similar, we deduce that IDR2 does not contribute significantly to the N-UPF2 interaction. Overall, it appears that the N-protein engages UPF2 in a multipartite interaction, with binding sites located within IDR1, the linker and DD but not in the RBD. We hypothesize that no individual site is sufficient for binding but a combination of two sites mediates an interaction with UPF2.

To test this hypothesis and to discern the contributions of IDR1 and the linker of N in binding to UPF2, we generated two variants of the N-protein where the 67-residue long linker connecting the RBD and DD was replaced by a short 12-residue stretch consisting of glycine, serine and alanine residues (GSA-linker). The length and position of the linker was based on available high-resolution X-ray crystal structures of the RBD and DD of N as well as a multiple sequence alignment of the nucleocapsid proteins from four betacoronaviruses (Supplementary figure S2). The first of these N variants included IDR1 in addition to the RBD and DD (referred to as N_IDR1-core_) while the second variant lacked IDR1 and comprised only the structured core domains (N_core_, Figure 1A). As the inter-domain linker is prone to proteolysis (55), the modified N-proteins lacking the long linker have a higher proteolytic stability than full-length N. GST-pulldown assays with these N variants showed that replacing the long linker with the short GSA-linker in N_IDR1-core_ did not compromise its binding to UPF2 (Figure 1D, lane 8). However, deletion of IDR1 in addition to removal of the long linker led to reduced binding (Figure 1D, lane 9), supporting our previous observations that a combination of any two binding sites on the N-protein is sufficient for mediating a strong interaction with UPF2. Correspondingly, an N variant containing only the IDR1 binding site (N_IDR1-RBD_) shows weak binding to UPF2 (Supplementary figure S1C).

**Figure 2.**
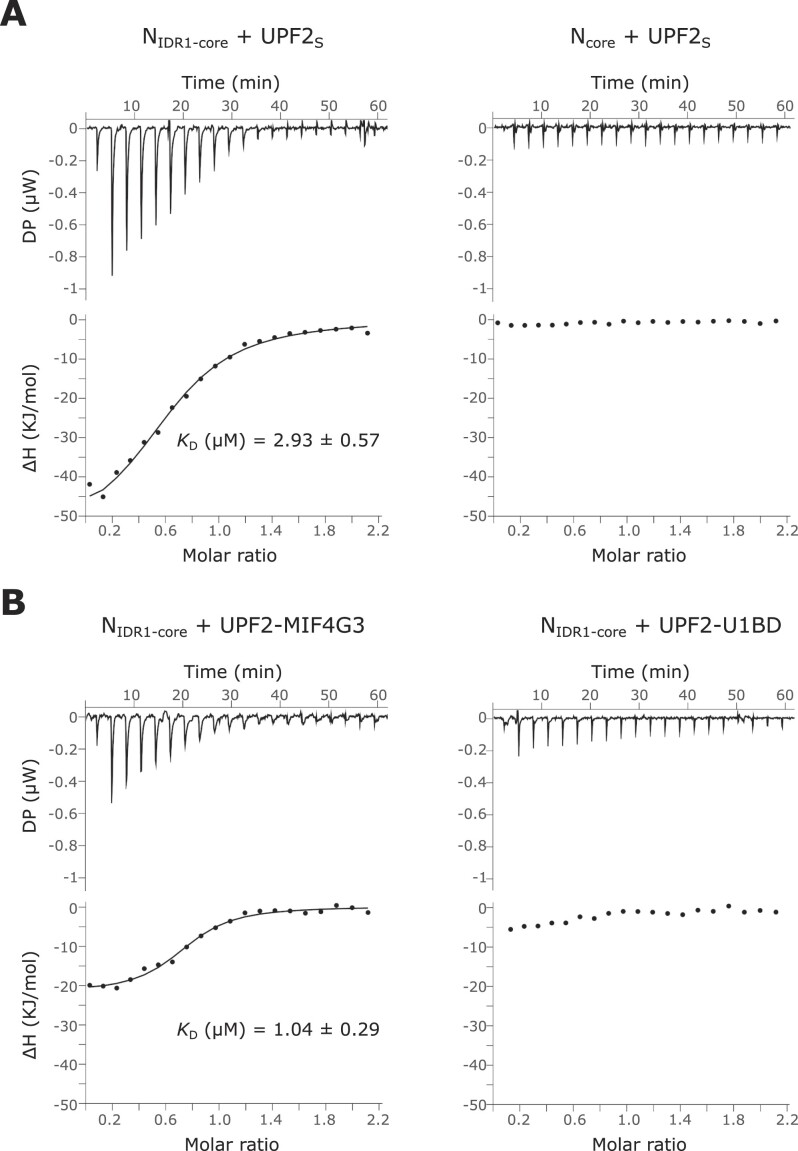
The MIF4G3 domain of UPF2 stably associates with an N variant comprising two of the three identified binding motifs. (**A**) Isothermal titration calorimetry (ITC) experiments of UPF2_S_ with N_IDR1-core_ (left panel) and N_core_ (right panel). The dissociation constant (*K*_D_) calculated from the binding isotherm is shown wherever applicable. Deletion of IDR1 in addition to the inter-domain linker abrogates binding of N to UPF2. (**B**) ITC experiments of N_IDR1-core_ with the MIF4G3 domain (left panel) and the U1BD (right panel) of UPF2. Removal of the U1BD does not impact binding of UPF2 to N (compare with left panel of Figure 2A), consistent with the observation that the U1BD shows no appreciable affinity for N. All N variants are considered as a dimer in this and subsequent experiments (see Supplementary figure S3A).

We used isothermal titration calorimetry (ITC) for a quantitative assessment of the interaction of UPF2 with N. As the full-length proteins are not stable over the duration of the measurements, we used the N_IDR1-core_ and UPF2_S_ proteins for ITC experiments. Furthermore, Nfl has a strong tendency to oligomerize and form unspecific higher-order assemblies in solution, precluding its use in ITC. Deletion of the long linker yielded a stable dimer of the resultant N-protein, N_IDR1-core_ (Supplementary figure S3A). The N_IDR1-core_ protein binds UPF2_S_ with an affinity of 2.9 μM (Figure 2A, left panel). Surprisingly, we did not detect any binding of N_core_ (lacking IDR1 and the linker) to UPF2_S_ in ITC despite observing a weak interaction in GST-pulldown assays (Figure 2A, right panel). Our results suggest that the three binding sites, IDR1, linker and DD, form a composite binding interface and at least two of the three sites are essential for N to stably engage UPF2.

Our observations with the different N variants in ITC prompted us to further investigate the binding of N to the different UPF2 domains using ITC. GST-pulldown assays described above indicate that the MIF4G3 domain of UPF2 is the primary binding site for the N-protein while additional weak interactions are mediated by the U1BD. Using ITC, we found that the UPF2-MIF4G3 domain alone bound the N-protein with an affinity comparable to that of UPF2_S_ (Figure 2B, left panel). However, no binding of the U1BD to the N_IDR1-core_ was detected (Figure 2B, right panel). Conversely, addition of UPF1_CH_ (which binds the U1BD) to UPF2_S_ did not influence the binding affinity of N for UPF2 (Supplementary figure S3B). Taken together, our results indicate that the interaction between N and UPF2 is driven predominantly by the MIF4G3 domain of UPF2.

### SARS-CoV-2 N engages the RNA helicase UPF1 via UPF2 and modulates its catalytic activity

**Figure 3.**
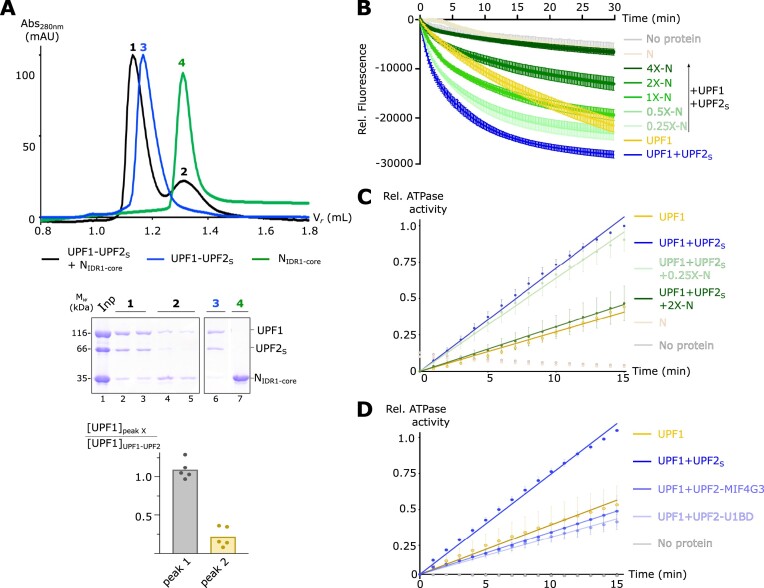
Indirect association of N with UPF1 inhibits its catalytic activity. (**A**) Analytical size-exclusion chromatography (SEC) depicting formation of a ternary complex of UPF1, UPF2_S_ and N_IDR1-core_ (top panel). The terms Abs and V*_r_* refer to absorbance (at 280 or 260 nm, as indicated in the figure) and retention volume, respectively, in this and all other figures. The exclusion volume of the column is 0.8 ml. SDS-PAGE analysis of the peak fractions visualized by Coomassie-staining, and a quantitative comparison of the relative amounts of UPF1 (normalized with respect to UPF1 in the UPF1-UPF2 complex) are shown in the middle and bottom panels, respectively. Interaction of N with the UPF1-UPF2 complex is sub-stoichiometric and leads to release of a small amount of UPF1 from the complex. (**B**) Fluorescence-based nucleic acid-unwinding activity measurements of a mixture of UPF1 and UPF2_S_ in presence of increasing concentrations of N (0.25- to 4-fold excess over UPF1). The data and the associated error bars represent the mean and standard deviation of three independent experiments. Technical duplicates were performed for each experiment. Controls without any protein and with N alone were included to monitor the stability of the RNA:DNA hybrid over the course of the experiment. The unwinding activity of UPF1 alone is shown for comparison. A low amount of N is sufficient to initiate inhibition of UPF1 unwinding activity, which is completely blocked in presence of high amounts of N. (**C**) RNA-dependent ATPase activity of a mixture of UPF1 and UPF2_S_ in the presence of low (0.25-fold of UPF1) and high (2-fold excess over UPF1) concentrations of N. An enzyme-coupled phosphate detection assay was used to measure ATPase activity. The data and the associated error bars represent the mean and standard deviation of three independent experiments. Technical duplicates were performed for each experiment. As with the unwinding activity measurements, a low amount of N slightly inhibits UPF1 ATPase activity while a higher amount shows more robust inhibition. (**D**) RNA-dependent ATPase activity of UPF1 in presence of different variants of UPF2 (added in 2-fold excess over UPF1). Although a UPF2 variant comprising the MIF4G3 and the U1BD domains strongly stimulates the ATPase activity of UPF1 (UPF2_S_, dark blue trace), these domains are not capable of activating UPF1 on their own. Experimental setup and data presentation are as described above in (C).

As the contribution of the U1BD to the N-UPF2 interaction appeared to be negligible, we proposed that UPF2 simultaneously engages UPF1 and N and thereby act as an adaptor to bridge the two proteins. To test this, we performed an analytical size-exclusion chromatography (SEC) assay using the N_IDR1-core_ protein, UPF2_S_ and UPF1. As predicted, UPF2 can simultaneously bind UPF1 and the N-protein to form a ternary complex, indicated by its lower retention volume compared to that of the UPF1–UPF2 complex (Figure 3A, top panel, compare black and blue traces). However, despite addition of an equimolar amount of N, the association of N with the UPF1–UPF2 complex is sub-stoichiometric, as indicated by the weaker intensity of N on SDS-PAGE and the second peak (peak 2) containing free N. Furthermore, it appears that a small fraction of UPF1 is also released from the complex as some UPF1 protein migrates at a higher retention volume, independent of the ternary complex (Figure 3A, peak 2 and lanes 4–5 of top and middle panels, respectively; the corresponding quantification of UPF1 levels in lanes 2 and 4 is shown in the bottom panel). It appears that although N can associate with UPF1 via UPF2, binding of N to UPF2 likely perturbs the UPF1-UPF2 interaction. Since the ATPase and nucleic acid-unwinding activities of UPF1 are stimulated upon binding UPF2, we hypothesized that the presence of N would impact UPF1 activity. To this end, we set up a nucleic acid-unwinding assay based on previous reports by Fritz and co-workers (56). Briefly, a 93 nucleotide (nt) long RNA strand was annealed at its 3′-end to an 18-nt complementary DNA strand labelled with a fluorophore at its 5′-end to create a fluorescence-labeled RNA:DNA duplex. Translocation of UPF1 on the RNA strand in an ATP-dependent manner resulted in unwinding of the duplex and release of the fluorescent DNA, which was efficiently captured and quenched by a Black hole quencher (BHQ1)-labelled trap strand complementary to the DNA. The resultant decrease in fluorescence was quantified as a measure of UPF1 activity (Figure 3B, yellow trace). Addition of UPF2_S_ to the reaction led to a rapid decrease in fluorescence, consistent with its role in stimulating UPF1 catalytic activity (Figure 3B, compare yellow and blue traces). Addition of an equimolar amount of N to the mixture of UPF1 and UPF2_S_ led to a significant reduction of UPF1 unwinding activity. The extent of inhibition by N was proportionate to the amount of N-protein added to the reaction (Figure 3B, green traces). As little as $\frac{1}{4}$th the molar amount of N with respect to UPF1 (molar ratio of N:UPF1 is 1:4) was sufficient to mediate a small reduction in unwinding activity (Figure 3B, pale green trace). RNA-dependent ATPase assays of a mixture of UPF1-UPF2_S_ in the presence of varying amounts of N showed a similar trend, with a small reduction in activity upon addition of limiting (0.25×) amounts of N-protein and robust inhibition in presence of excess (2×) N (Figure 3C). Taken together, our observations show that interaction of N with UPF2 impairs the ability of UPF2 to stimulate UPF1 catalytic activity. At first glance, this appears incongruous as N and UPF1 bind two distinct domains of UPF2, the MIF4G3 and U1BD, respectively, and activation of UPF1 by UPF2 is a consequence of a conformational change induced upon interaction. However, our ATPase assays show that the U1BD of UPF2 alone cannot activate UPF1. The MIF4G3 domain is essential for robust activation of UPF1, although it does not exert this effect alone either (Figure 3D). We suggest that binding of the U1BD of UPF2 to UPF1 positions the UPF2-MIF4G3 domain close to the helicase core of UPF1, which has a positive impact on UPF1 catalytic activity. Binding of N to the UPF2-MIF4G3 domain interferes with this effect and prevents complete activation of UPF1 by UPF2.

### SARS-CoV-2 N displaces UPF1 from RNA at higher concentrations and indirectly inhibits its catalytic activity

**Figure 4.**
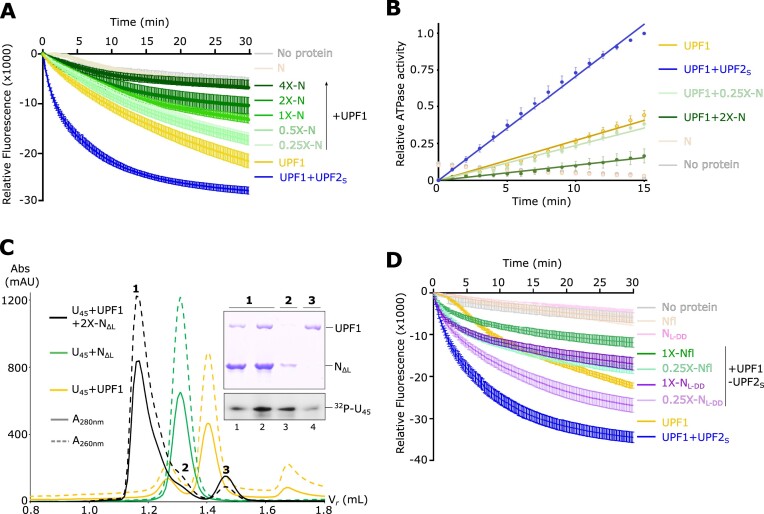
High concentrations of N impede UPF1 catalytic activity by displacing it from RNA. (**A**) Nucleic acid-unwinding activity of UPF1 in presence of increasing concentrations of N (0.25- to 4-fold excess over UPF1). The experiment, including controls, was conducted as described in Figure 3B. The unwinding activity of a mixture of UPF1 and UPF2_S_ is shown for comparison. In absence of UPF2, N inhibits UPF1 activity only at high concentrations. (**B**) RNA-dependent ATPase activity of UPF1 in presence of low (0.25-fold) and high (2-fold excess) concentrations of N. As observed with unwinding activity measurements, inhibition is only achieved upon addition of high amounts of N. (**C**) Analytical SEC experiments of UPF1 and U_45_ RNA in the presence of 2-fold excess of N_ΔL_. An overlay of the chromatogram of the U_45_-UPF1-N mixture (black) with those of U_45_-N (green) and U_45_-UPF1 (yellow) are shown in the top panel. The bottom panels show the corresponding SDS- and urea–PAGE analyses of peak fractions of the U_45_-UPF1-N chromatogram. Proteins were visualized by Coomassie staining and RNA was detected by radiolabeling with ^32^P followed by phosphorimaging. Addition of an excess of N to a UPF1–RNA mixture leads to higher occupancy of N on RNA (peaks 1 and 2) and a concomitant release of UPF1 from RNA (peak 3). A quantitative comparison of the relative amounts of UPF1 released upon addition of equimolar and 2-fold excess of N is shown in Supplementary figure S4C. (**D**) Effect of addition of sub-stoichiometric (0.25×) and equimolar (1×) amounts of N_L-DD_ on the nucleic acid-unwinding activity of UPF1 in complex with UPF2_S_. Reactions carried out in the presence of Nfl are shown for comparison. N_L-DD_ does not bind RNA but interacts with UPF2 and inhibits UPF1 in the UPF1–UPF2 complex. No inhibition is observed in the absence of UPF2 (see Supplementary Figure S4D).

The observations described above suggest that the influence of N on the catalytic activity of UPF1 can only be exerted through UPF2. However, activity assays of UPF1 alone showed that when present in excess, N can directly inhibit both unwinding as well as the ATPase activities of UPF1 (Figures 4A and B). Unlike with UPF1–UPF2, low amounts of N do not significantly modulate the catalytic activity of UPF1 alone. Although N specifically binds sequence and structural elements at the 5′-UTR of the SARS-CoV-2 genomic RNA (57,58), *in vitro* it can bind a generic homopolymeric sequence (such as poly-U) and mediate liquid-liquid phase separation (59). Given that UPF1 also binds RNA without any sequence specificity, we asked if N and UPF1 compete for binding RNA and if this influences the catalytic activity of UPF1. We first used fluorescence anisotropy to determine the binding affinity of N for a 12-mer poly-U RNA (U_12_) labelled with 6-FAM at its 5′-end. The binding affinity of Nfl as well as N_ΔL_ for RNA is comparable to that previously determined for UPF1 (*K*_D_ of ∼ 50 nM for UPF1-RNA vs 82 nM for Nfl-RNA and 45 nM for N_ΔL_-RNA, Supplementary figure S4A). Analytical SEC assays of a mixture of UPF1, a 45-mer poly-U RNA (U_45_) and 2-fold molar excess of N_ΔL_ showed that N and UPF1 can co-occupy the U_45_ RNA, as indicated by a single peak containing both proteins as well as the RNA (Figure 4C, peak 1 and lanes 1–2 of SDS gel). N and UPF1 do not form a stable complex in the absence of RNA (Supplementary Figure S4B). The retention volume of the N-UPF1-RNA peak (peak 1) is lower than that of the peaks corresponding to the N-RNA and UPF1-RNA complexes, confirming that it represents a protein–RNA complex comprising both N and UPF1. However, the amount of N in peak 1 is higher than that of UPF1, based on the intensities of the bands on the Coomassie-stained gel. Additionally, we observed an additional peak (peak 3) corresponding to free UPF1, which has a lower absorbance at 260 nm than at 280 nm and correspondingly, a lower amount of RNA (Figure 4C, peak 3 and lane 4 of SDS gel). The amount of free UPF1 obtained depends on the fold-excess of N-protein added, where excess N stimulates a greater release of UPF1 from RNA (Supplementary Figure S4C). It appears that although N binds RNA with an affinity comparable to UPF1 and there is no direct competition for RNA binding, excess N can impede RNA-binding by UPF1, rendering it incapable of translocating on RNA and mediating its ATPase and unwinding activities.

Our results point to two different modes of inhibition of UPF1 catalytic activity by the N-protein: an indirect mode via its interaction with UPF2, which interferes with UPF1 activation, and a direct mode by perturbing UPF1 RNA binding. This raises the question of whether the N-UPF2 interactions play a significant role in inhibiting UPF1, particularly at higher concentrations of the N-protein. To address this, we tested the ability of a variant of N spanning the inter-domain linker and the dimerization domain (N_L-DD_) to inhibit UPF1 unwinding activity. N_L-DD_ lacks the RBD and shows a drastic reduction in RNA-binding affinity in comparison to Nfl (Supplementary Figure S4A) but mediates strong interactions with UPF2 (Figure 1D, lane 7). We found that N_L-DD_ inhibits UPF1 unwinding activity in the presence of UPF2 in a concentration-dependent manner, albeit to a lower extent than Nfl (Figure 4D, compare purple and green traces). We hypothesize that as N_L-DD_ cannot bind RNA, it is incapable of hindering UPF1 RNA-binding, and therefore does not inhibit UPF1 catalytic activity via this mode. Accordingly, N_L-DD_ does not inhibit UPF1 unwinding activity in the absence of UPF2, even at a higher concentration (Supplementary Figure S4D). Taken together, these observations provide mechanistic evidence for the role of N-UPF2 interactions in the overall inhibition of UPF1 catalytic activity by the N-protein.

### Impact of SARS-CoV-2 N on host cell NMD targets

**Figure 5.**
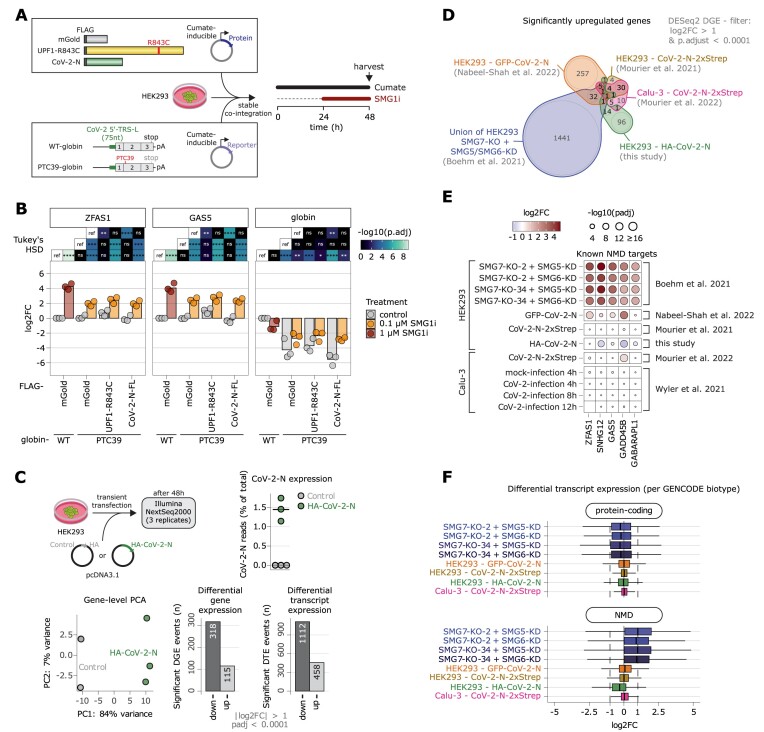
NMD is not substantially inhibited by N overexpression in human cell lines. (**A**) Experimental overview including schematic representations of the overexpressed FLAG-tagged proteins and beta-globin reporters. Expression of proteins and reporter was induced for 48 h with cumate, whereas treatment with different concentrations of SMG1i was performed for 24 h. (**B**) Probe-based quantitative RT-PCR analysis of GAS5 and ZFAS1 and SYBR-green-based analysis of globin expression levels in the respective overexpression and treatment condition normalized to the B2M reference. FLAG-mGold serves as negative control for protein expression and WT-globin as control reporter. Data points and means are plotted as log_2_ fold change (log_2_FC) (*n* = 3). Statistical analysis via Tukey's HSD was performed with adjusted p-value significance cutpoints 0.05 (*), 0.01 (**), 0.001 (***) and 0.0001 (****). (**C**) Overview of N overexpression RNA-Seq experiment, percentage of reads mapping to N, clustering of samples by PCA and differential expression analysis on gene- and transcript-level. (**D**) Overlap of significantly upregulated genes (cut-offs are indicated) in RNA-Seq data from four different CoV-2-N overexpression datasets and the union of four NMD-factor knockout/knockdown combinations. (**E**) Heatmap of DESeq2-derived log_2_ fold changes of five selected known NMD target genes in various RNA-Seq datasets. The size of points corresponds to the statistical significance. The CoV-2-N-2xStrep dataset is obtained from: https://www.medrxiv.org/content/10.1101/2021.05.06.21256706v2. (**F**) Boxplots of differential transcript expression determined by Swish and stratified by GENCODE biotype (protein-coding and nonsense-mediated decay).

Our biochemical and biophysical results suggest that the SARS-CoV-2 N-protein can potentially interfere with the proper cellular function of UPF1 in NMD. To test this hypothesis, we generated stably transfected Flp-In-T-REx-293 cells, which express N-terminally FLAG-tagged proteins, and a modified beta-globin NMD reporter (PTC39) or the corresponding wild type (WT) reporter, both in a cumate-inducible manner (Figure 5A). The modified beta-globin reporters are derived from the established beta-globin NMD reporters (60), and were generated by cloning the 75-nt long SARS-CoV-2 5′ transcription-regulatory leader sequence (TRS-L) upstream of the globin sequence to mimic the viral sg-mRNAs and potentially recruit N protein to these reporters. The fluorescent protein mGold served as a negative control and a previously reported dominant negative UPF1 mutant (R843C) as an additional control (61). After 48 h of cumate induction, western blot analysis confirmed the expression of all proteins (Supplementary Figure S5A).

We observed strongly reduced PTC39-globin mRNA levels compared to the WT-globin in qPCR experiments, indicating that the NMD reporter mRNAs are degraded (Figure 5B). However, we could not detect significant increase in PTC39-globin levels upon expression of UPF1-R843C or CoV-2-N. When investigating the expression levels of two known endogenous NMD targets (ZFAS1 and GAS5; snoRNA host genes) (62), we observed modest upregulation in the UPF1-R843C condition, whereas N showed no effect (Figure 5B). We next asked if partial NMD inhibition by treating cells with low concentrations of SMG1 kinase inhibitor (SMG1i) could reveal potential additive inhibitory effects, as observed in a recent study (https://www.biorxiv.org/content/10.1101/2024.04.15.589496v1). By quantifying ZFAS1 and GAS5 levels, we first established that treatment with high concentrations (1 μM) of SMG1i for 24h strongly inhibited NMD (Figure 5B). In contrast, low concentrations (0.1 μM) of SMG1i only partially upregulated ZFAS1, GAS5 or PTC39-containing globin reporter mRNA. Also in these conditions, we could not detect any additive inhibitory effect on NMD activity by expression of UPF1-R843C or CoV-2-N. Analysis of a well-studied NMD-targeted alternative splicing isoform (SRSF2) by end-point PCR confirmed the general observations of our qPCR analyses (Supplementary Figure S5B).

To analyze the transcriptome-wide impact of N expression on NMD activity, we performed RNA-Seq of transiently transfected HEK293 cells, expressing either HA-tagged CoV-2-N or empty vector as control (Figure 5C). Of note, expression of N and interaction with cellular UPF2 was confirmed by co-IP and western blot analyses (Supplementary Figure S5C). About 1.5% of total RNA-Seq reads mapped to the N sequence, indicating strong expression levels (Figure 5C and Supplementary Figure S5C). The N expressing samples separated well from control in a principal component analysis (PCA) and we surprisingly detected primarily significantly downregulated genes and transcripts (Figure 5C). To put these results into broader perspective, we analyzed publicly available RNA-Seq datasets (36,37). Since NMD inhibition is expected to lead to the accumulation of NMD-targeted transcripts, we compared the significantly upregulated genes among our own dataset, three different N-overexpressing conditions (two in HEK293, one in Calu-3 cells) and in relation to a strong NMD inhibition (SMG7 knockout + SMG5/SMG6 knockdown, (35)). Overall, the number of overlapping genes between the different studies is low and only 1 gene is consistently found as significantly upregulated in all conditions (IFI44L; Figure 5D and Supplementary Figure S5D). This gene is implicated in the defense response to viruses (gene ontology term GO:0051607) and has at least one NMD-annotated transcript in the GENCODE database. When investigating five known commonly upregulated NMD targets (including ZFAS1 and GAS5), we do not find compelling evidence that overexpressed N protein acts as a broad NMD inhibitor (Figure 5E). Furthermore, no significant upregulation of those NMD targets could be observed in RNA-Seq data from Calu-3 cells infected with SARS-CoV-2 for different timepoints (4–12 h, Figure 5E, (38)). Next, we analyzed the differential transcript expression (DTE) and asked whether NMD-annotated transcripts are preferentially upregulated. Whereas this is very apparent for the strongly NMD-inhibited SMG7-KO + SMG5-KD conditions, N overexpression did not result in substantial NMD-isoform accumulation (Figure 5F and Supplementary Figure S5E). Taken together, our analyses of various overexpression studies could not identify a detectable NMD-inhibitory role of SARS-CoV-2 N in cultured human cell lines.

## Discussion

The influence of NMD factors on the replication of positive-sense RNA viruses has been previously investigated in detail. Depletion of NMD factors leads to increase in the efficiency of replication and propagation of the Semliki Forest virus (SFV) (63). The viral genomic RNA as well as sub-genomic mRNA of MHV was shown to be recognized by the NMD pathway and consequently, stabilized upon inhibition of NMD (18). As with SFV, depletion of NMD factors leads to an enhancement in MHV replication. It follows that these viruses must therefore develop mechanisms to inhibit NMD in order to facilitate their replication and propagation. The capsid protein of SFV and the nucleocapsid protein (N) of MHV were shown to inhibit the NMD pathway, suggesting a direct intervention of the viral proteins in this decay mechanism (18,64). In this study, we show for the first time, a direct interaction of the nucleocapsid protein of a betacorona virus with factors involved in NMD. We find that the SARS-CoV-2 N-protein can associate with the NMD core factor UPF2, and through it, the RNA helicase UPF1, accounting for the observed interaction between SARS-CoV-2 N and UPF1 in a high-throughput interactome study.

The N-protein uses a combination of IDRs and a structured domain to interact with the MIF4G3 domain of UPF2. IDRs are often involved in mediating protein-protein interactions due to their intrinsic flexibility and their ability to adopt an ensemble of different conformations. They can adopt a distinct fold upon binding a structured domain or can engage in fuzzy interactions where the conformation of the IDR in the complex remains heterogenous. IDRs can also interact with unstructured proteins/region to form complexes of diverse stabilities (65). The inter-domain linker and IDR1 of N together with its C-terminal dimerization domain form a composite binding surface for UPF2. While the dimerization domain is highly conserved across the SARS, MERS and MHV nucleocapsid proteins, sequence similarity across IDR1 and the inter-domain linker is low. Interestingly, the serine/arginine-rich sequences within the linker that are sites of phosphorylation are present in all the N-proteins analysed (Supplementary figure S2). Phosphorylation of SARS-CoV-2 N was shown to modulate formation of biomolecular condensates, suggesting a role in self-association of the N-protein (66). Correspondingly, the linker was shown to play an important role in the higher order oligomerization of N, which is critical for packaging the viral genome and stabilizing the genomic RNA. Of the three interfaces of N that we identified as UPF2-interacting regions, the linker appears to have the least contribution to binding as its removal does not significantly reduce binding to UPF2 (Figure 1D). It is therefore plausible that the additional interactions mediated by the linker with host cell proteins such as UPF2 are weaker than those forged by IDR1 and the DD as it is already involved in *cis*-intermolecular interactions.

UPF2 plays multiple roles in NMD. It directly binds UPF1 and is believed to engage the helicase in a large assembly referred to as the decay-inducing complex (DECID) that involves the exon junction complex (EJC) and the eukaryotic release factors (ERFs) (54,67–69). It was also thought to facilitate phosphorylation of UPF1 which is crucial for NMD in metazoans. Additionally, binding of UPF2 to UPF1 mediates its release from RNA and stimulates its catalytic activity, which is essential for NMD (31). The modular domain arrangement of UPF2 enables these functions as it uses distinct domains to associate with UPF3-EJC on one hand, and with UPF1 on the other. Binding of UPF3 to the UPF2-MIF4G3 domain does not impact the association of UPF1 with UPF2 (50). Similarly, concomitant binding of UPF2 to STAU1 and UPF1 in the SMD pathway allows for activation of UPF1 within the ternary complex (52). In comparison to these assemblies, the interaction of N to UPF2 is rather unusual. The N-protein binds the MIF4G3 domain of UPF2 and can form a ternary complex, albeit sub-stoichiometric, with UPF1 and UPF2. However, instead of activation of UPF1 as is typically observed in the other ternary complexes mentioned above (UPF1–UPF2–UPF3 and UPF1–UPF2–STAU1), we observe a strong inhibition of UPF1 catalytic activity. We speculate that the MIF4G3 surface that binds N is distinct from the one that binds UPF3 and STAU1. Fittingly, our preliminary data show that UPF2 can simultaneously bind N and UPF3 in analytical SEC (Supplementary Figure S5F). Given that the UPF2-MIF4G3 domain has a strong positive influence on UPF1 catalytic activity (Figure 3D), it is possible that binding of N to it negates this effect and as a result, UPF1 is no longer activated by UPF2 even though it remains bound through the U1BD.

In addition to the effect mediated upon binding of N to UPF2, we observe a robust inhibition of UPF1 catalytic activity by N alone. Unlike in the presence of UPF2, this inhibitory effect is only observed with an excess of the N-protein. We attribute this to high amounts of N coating the RNA and blocking access of UPF1 to the RNA (see also Figure 4C). The combined effect of both modes of inhibition allows for efficient dampening of UPF1 catalytic activity, while ensuring redundancy in the process.

**Figure 6.**
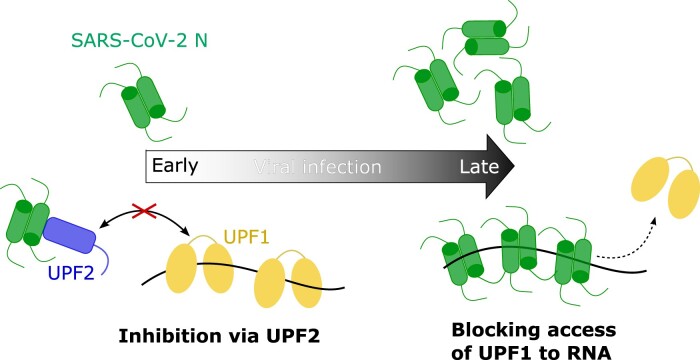
A mechanistic model for inhibition of UPF1 catalytic activity on SARS-CoV-2 RNA by N-protein. The amount of N in host cells continuously increases with progression of infection. Despite robust inhibition of UPF1 catalytic activity by SARS-CoV-2 N, host cell NMD targets remained unaffected by expression of the N-protein. The NMD inhibitory effect of MHV N on viral RNA allows us to speculate a similar role for SARS-CoV-2 N in protecting its own genomic and sg-mRNA.

Given the high degree of sequence conservation between the SARS-CoV-2 and MHV N-proteins and previous reports of the NMD inhibitory function of MHV N with respect to viral RNA, we extrapolate our *in vitro* biochemical observations on inhibition of UPF1 catalytic activity by N to propose the following model for the interplay of SARS-CoV-2 N and NMD (Figure 6). At early stages of viral infection, the levels of N-protein in the host cell are low. At this stage, the genomic RNA needs to be efficiently replicated and the sg-mRNAs need to be produced in large amounts to generate the structural proteins necessary for viral packaging and propagation. Therefore, it is imperative that the genomic RNA as well as the sg-mRNA are protected from degradation by the NMD pathway and are adequately stabilized. We speculate that small amounts of N present at this stage binds UPF2 and prevents it from activating UPF1, thereby minimizing decay of the viral RNA. As infection progresses, so does the accumulation of N in the host cell. The amount of N-protein increases by approximately 11-fold within 24 h post-infection (70). Large amounts of N coat the viral RNA and can prevent binding of UPF1 and consequently, the onset of NMD (Figure 6). Despite the potent inhibitory effect of N observed *in vitro*, our RNA-seq analysis in HEK293 cells does not show transcriptome-wide stabilization of cellular NMD targets upon expression of N-protein. This is particularly interesting as the MHV N-protein was previously shown to stabilize a luciferase reporter that mimics an MHV sg-mRNA. Yet, we find that insertion of the SARS-CoV-2 TRS-L sequence in the 5′-UTR is not sufficient to stabilize an NMD reporter in the presence of N, suggesting that other features of the viral RNA might play a role in recruitment of N (57). Global shutdown of NMD would be fatal for host cells and therefore detrimental to viral replication and propagation. It is therefore intuitive that especially at early stages of viral infection, the N-protein does not block the general decay of cellular NMD targets. The underlying mechanism of how SARS-CoV-2 N protects viral RNA without interfering with host cell NMD targets remains a question for future studies.

## Supplementary Material

gkae829_Supplemental_File

## Data Availability

All original data will be made available upon request. RNA-Seq data generated in this study have been deposited at BioStudies/ArrayExpress under accession number E-MTAB-14342 (https://www.ebi.ac.uk/arrayexpress/experiments/E‐MTAB‐14342) and are publicly available as of the date of publication.
