# Supplementary material for: Modulation of UPF1 catalytic activity upon interaction of SARS-CoV-2 Nucleocapsid protein with factors involved in nonsense mediated-mRNA decay
Source: Nucleic Acids Res. 2024 Oct 3;52(21):13325–39. doi: 10.1093/nar/gkae829 (PMC11602160; doi:10.1093/nar/gkae829)
Supplement: gkae829_Supplemental_File [file gkae829_supplemental_file.pdf]

Supplemental Information

Modulation of UPF1 catalytic activity upon interaction of SARS-CoV-2 Nucleocapsid protein with factors involved in nonsense mediated-mRNA decay

Megha Mallick, Volker Boehm, Guangpu Xue, Mark Blackstone, Niels H. Gehring and Sutapa Chakrabarti

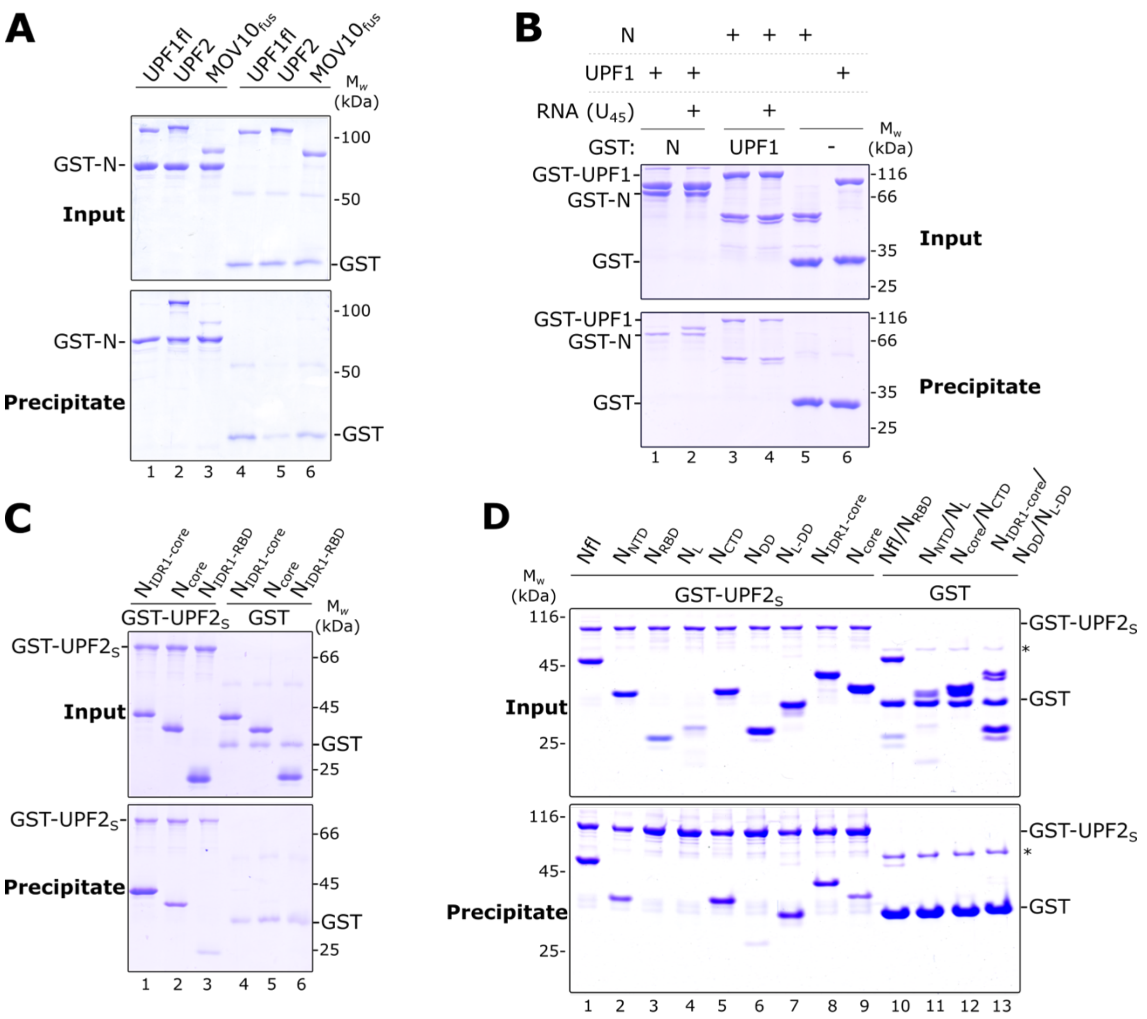

Supplementary figure 1. The SARS-CoV-2 Nucleocapsid (N) protein directly interacts with the core NMD factor UPF2. A) GST-pulldown assay of full length UPF1 (UPF1fl), UPF2, and MOV10$_{fus}$ with full-length GST-N as a bait. GST was used as a negative control in all such assays. The top and bottom panels depict input and precipitate, respectively, in this and all the other GST-pulldown assays. GST-N binds to UPF2 but not UPF1fl or MOV10$_{fus}$. **B)** GST-pulldown assay to test the N-UPF1 interactions in the absence and presence of RNA (poly-U). Binding between GST-N and UPF1 was observed only upon addition of RNA. GST-UPF1 showed weak interactions with N, which were not enhanced in the presence of RNA. **C)** GST-pulldown assay to compare binding of the IDRs and the structured domains of N to UPF2. GST-UPF2$_S$ is used as a bait and N$_{IDR1\text{-}core}$, N$_{core}$ and N$_{IDR1\text{-}RBD}$ as preys. Variants of N containing only one of the three identified binding motifs (IDR1, linker and DD), such as N$_{core}$ and N$_{IDR1\text{-}RBD}$, show weaker binding to UPF2 in comparison to N$_{IDR1\text{-}core}$, which contains two of three binding motifs. We conclude that no single binding site of N can mediate a strong interaction with UPF2: a combination of any two binding sites is necessary for a stable N-UPF2 interaction. **D)** A complete gel corresponding to Figure 1D, including negative controls using GST as a bait. Domain organization of the N-constructs are shown in Figure 1A. The asterisk (*) indicates a contaminant.

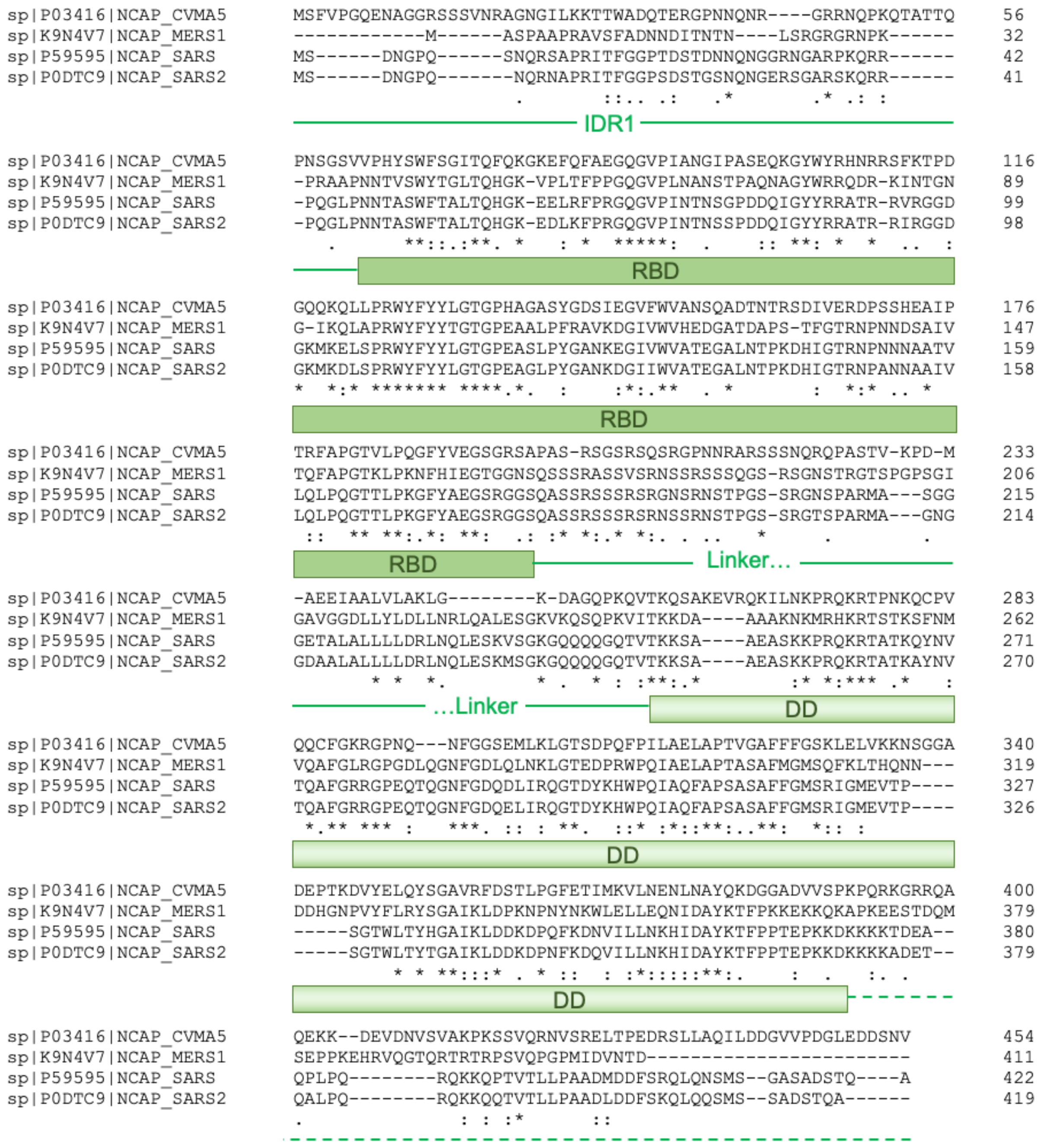

Supplementary figure 2. Multiple sequence alignment using Clustal Omega (Clustal O 1.2.4) with N-protein sequences from different coronaviruses (P03416: Murine Hepatitis Virus, K9N4V7: MERS, P59595: SARS-CoV and P0DTC9: SARS-CoV-2, acquired from Uniprot: https://www.uniprot.org/). The sequence similarities across species are indicated as follows: * - fully conserved, : - strongly similar, . – weakly similar. The domain organization of SARS-CoV-2 N is indicated below the alignment. Shaded boxes denote structured domains and lines depict intrinsically disordered regions (IDRs) flanking the domains. Solids lines indicate the N-terminal IDR and the inter-domain linker (labeled) which were identified to contain UPF2-binding sites in this study. Sequence conservation is highest among amino acids within the structured regions and considerably lower across the IDRs.

A

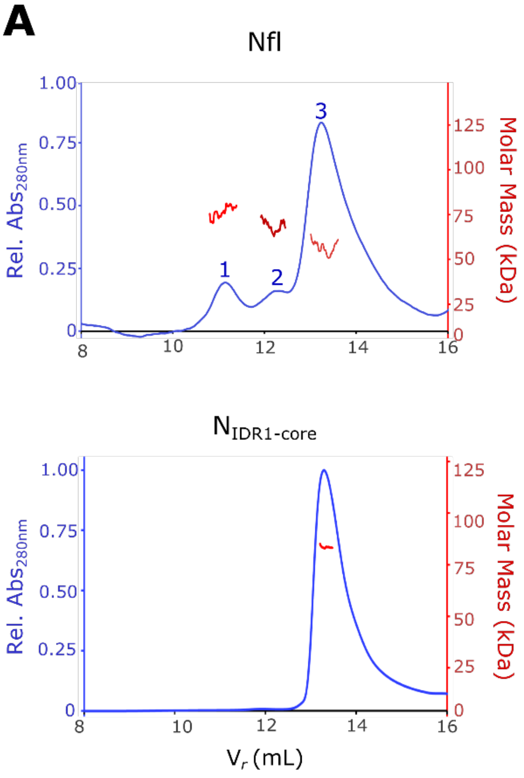

Theoretical M_w (kDa)	Experimental M_w (kDa)
Nfl<br>Monomer = 45.6<br>Dimer = 91.2	Peak 1 = 80.1 ± 9.2<br>Peak 2 = 72.8 ± 9.2<br>Peak 3 = 58.3± 5.7
$N_{IDR1\text{-}core}$<br>Monomer = 36.7<br>Dimer = 73.4	Peak = 83.6 ± 1.5

B

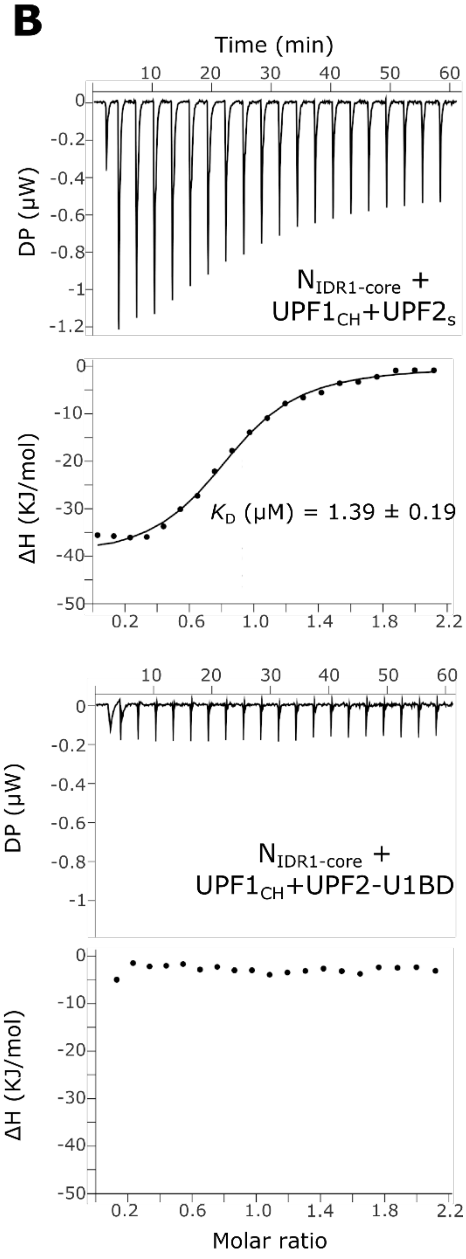

Supplementary figure 3. A) Size Exclusion Chromatography-Multi-angle Light Scattering (SEC-MALS) analysis to determine the oligomerization state of full-length N (Nfl) and $N_{IDR1\text{-}core}$ and the molar mass (kDa) across the observed peaks. The experimentally-obtained values are indicated in the table below, along with the theoretical molar mass (calculated using the Expasy Protparam tool) for distinct oligomerization states of each N construct. Nfl exists as a mixture of indistinct oligomers in solution whereas $N_{IDR1\text{-}core}$, lacking the long inter-domain linker, forms a homogenous dimer. **B)** Isothermal titration calorimetry (ITC) experiments of $N_{IDR1\text{-}core}$ with complexes of $UPF1_{CH}$ and UPF2 ($UPF2_S$ and UPF2-U1BD, top and bottom panels, respectively) show that addition of UPF1 does not influence the binding affinity of N to UPF2 (compare K_D derived from this experiment to that in Figure 2A).

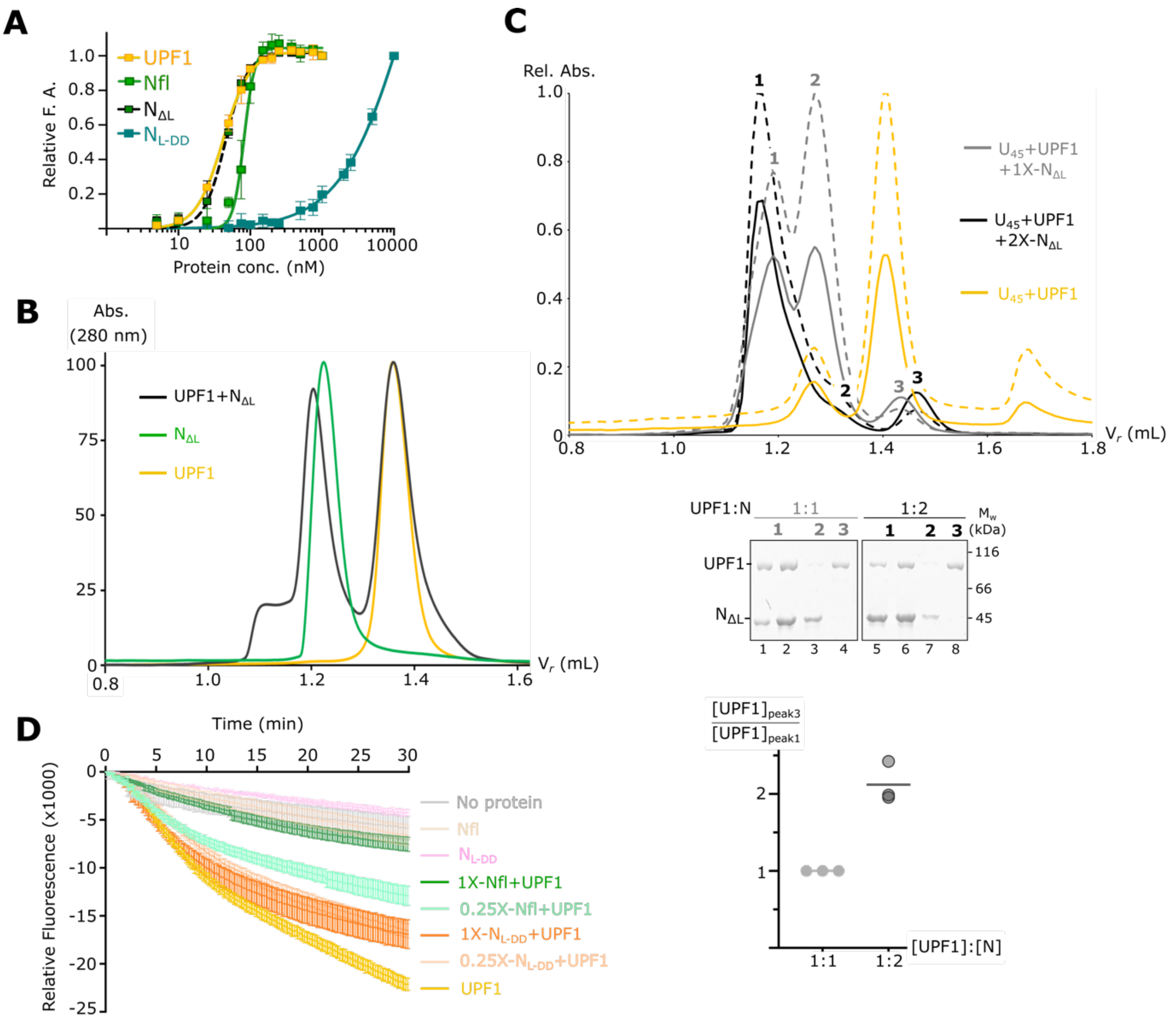

Supplementary figure 4. A) Fluorescence anisotropy measurements using a 5'-end 6-FAM labeled12-mer poly-U RNA (U_{12}) shows that UPF1 (yellow trace), Nfl (green trace) and $N_{\Delta L}$ (black dashed trace) have comparable binding affinities towards the RNA with a K_D of ~ 50 nM for UPF1-RNA, 82 nM for Nfl-RNA and 45 nm for $N_{\Delta L}$-RNA. $N_{L\text{-}DD}$ (teal trace) has a much lower binding affinity than Nfl and $N_{\Delta L}$ (> 16 μM). The data points and their error bars represent the mean and the standard deviation of at least 3 independent experiments. The K_D was determined by fitting the data points to an equation representing one-site specific binding with Hill slope using the program Prism 10. **B)** Analytical size exclusion chromatography (SEC) of an equimolar mixture of UPF1 and $N_{\Delta L}$ (black trace). SEC analysis of UPF1 and $N_{\Delta L}$ alone (yellow and green traces, respectively) are shown for comparison. No interaction is observed between the two proteins in the absence of RNA. **C)** Analytical SEC, and the corresponding SDS-PAGE analyses of mixtures of UPF1, a 45-mer poly-U RNA (U_{45}) and $N_{\Delta L}$. UPF1 and U_{45} are in equimolar amounts; $N_{\Delta L}$ is either added in equimolar amounts (grey trace) or in 2-fold excess to UPF1 (black trace). Solid and dashed lines refer to absorbance at 280 nm and 260 nm, respectively. Quantification of the bands corresponding to free UPF1 (lanes 4 and 8) is shown in the bottom panel. Although N and UPF1 can co-

occupy the RNA (lanes 1 and 5, corresponding to peaks 1 and 4), a small peak corresponding to RNA-free UPF1 is always observed (lanes 4 and 8, corresponding to peaks 3 and 6). Addition of a 2-fold excess of N leads to an approximately 2-fold increase in free UPF1 (bottom panel). **D)** Nucleic acid-unwinding activity of UPF1 in presence of low (0.25-fold of UPF1) and equal concentrations of Nfl (green traces) and $N_{L\text{-}DD}$ (orange traces). The experiment, including controls, was conducted as described in Figure 3B. In absence of UPF2, significant inhibition of UPF1 unwinding activity is achieved upon addition of equal concentrations of Nfl but not of $N_{L\text{-}DD}$.

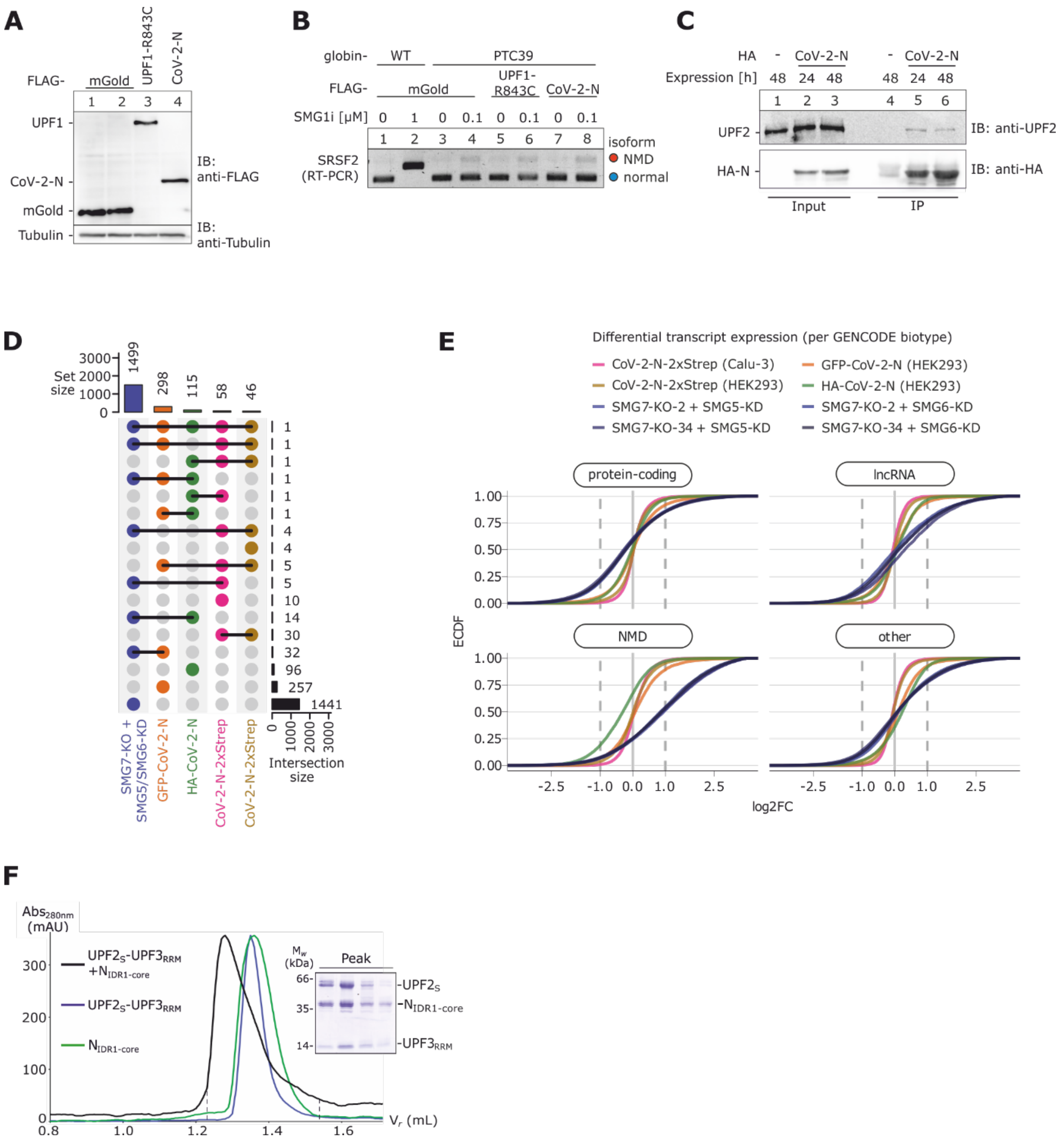

Supplementary figure 5. A) Western blot analysis of FLAG-tagged protein expression in HEK293 cells with Tubulin serving as loading control. **B)** End-point PCR analysis of SRSF2 isoforms in FLAG-tagged overexpression and SMG1i-treated conditions. **C)** Co-immunoprecipitation of HA-tagged CoV-2 N and cellular UPF2 from HEK293 lysates. **D)** UpSet plot showing the overlap of significantly upregulated genes in the indicated RNA-Seq datasets. **E)** Cumulative distribution function plots of differential transcript expression determined by Swish and stratified by GENCODE biotype. **F)** Analytical size-exclusion chromatography (SEC) shows that UPF2 binds N and UPF3 simultaneously. SDS-PAGE analysis corresponding to peak 1 of the black trace depicts formation of a ternary complex of $UPF2_S$-$UPF3_{RRM}$-$N_{IDR1\text{-}core}$. The terms Abs_{280nm} and V_r refer to absorbance at 280 nm and retention volume, respectively.

Supplementary Methods

Expression and purification of UPF1fl from a baculovirus system

Hi5 cells at a density of $0.8x10^6$ cells/mL were infected with a first generation virus encoding UPF1fl at a 1:1000 v/v ratio (virus:cells). Cells were harvested 68 hours post-infection by centrifugation at 800xg for 10 mins. The cell pellet was rinsed with 1X phosphate-buffered saline (PBS) and resuspended in lysis buffer A (50 mM Tris-HCl pH 7.5, 500 mM NaCl, 10 mM imidazole and 10 % glycerol) supplemented with 1 mM phenylmethylsulphonyl fluoride (PMSF) and 0.5 mg of DNase I. Cells were lysed by low-pulse sonication. All subsequent steps were conducted as described for the purification of *E. coli* expressed proteins in the main text.

SEC-MALS

To measure the absolute molar mass of the SARS-CoV-2 Nfl and $N_{IDR1\text{-}core}$ proteins, approximately 60 µg of protein at a concentration of 1 mg/ml was injected onto a Superdex 200 10/300 column (GE Healthcare) on an Agilent HPLC system coupled to miniDWAN TREOS (Wyatt Technology, Germany) and Refracto-Max520 (ERC) detectors. The protein was eluted using a thoroughly degassed running buffer containing 20 mM Tris-HCl, pH 8.0, 150 mM NaCl, 5% glycerol, 0.02% NaN_3 and 2 mM DTT at room temperature. The instrument was calibrated using bovine serum albumin as a reference. Data recording and analysis was performed using the ASTRA 6.1.4.25 software (Wyatt Technology, Germany).

Co-immunoprecipitation

For co-immunoprecipitation of UPF2 and N-protein, human embryonic kidney 293 (HEK293) cells were seeded in 6-well plates and grown to 75% confluence. 1 µg plasmid of pCDNA3.1-HA-SARS-CoV-2 N was transfected using Lipofectamine 2000 (Invitrogen). Cells were harvested 24- and 48-hours post-transfection, washed with 1 ml phosphate buffer saline (PBS) and lysed with 250 µl NET-G buffer (50 mM Tris-HCl pH 7.5, 150 mM NaCl, 0.1 % NP-40, 1 mM EDTA; pH 8.0, 1x complete Protease Inhibitor cocktail (Roche) and 10 % glycerol). 15 µl Protein A/G Agarose-PLUS (Santa Cruz Biotechnology:sc-2003) beads were prepared for each sample (control, 24 h and 48 h), washed with NET-G buffer and incubated with 10 µl anti-mouse-HA antibody (BioLegend, Catalogue# 901513) for 1 h at 4 °C, followed by washing with NET-G buffer. 10 µg of each cell lysate was removed as input, and lysates were incubated with washed antibody-bound beads for 16 hours at 4°C. Following extensive washing with NET-G buffer, bound proteins were eluted from the beads using 1X Laemmli buffer lacking reducing agents. Co-immunoprecipitation of UPF2 with SARS-CoV-2 N-protein was detected by western blot using anti-UPF2 antibody (Bethyl Laboratories, Catalogue#

A303-929A, dilution of 1:4000). The SARS-CoV-2 N-protein was detected using an anti-HA antibody (BioLegend, Catalogue# 901513, dilution of 1:2500).

Fluorescence anisotropy

For determination of the binding affinity of SARS-CoV-2 N for RNA, 5 nM of a 5'-end 6-FAM labeled 12-mer poly-U-RNA was mixed with increasing concentrations of Nfl and a modified stable construct of N-protein ($N_{\Delta L}$) in a binding buffer (20 mM HEPES pH 7.5, 100 mM NaCl, 1 mM $MgCl_2$ and 100 µg/ml BSA) and incubated at 25 °C for 1 h in the dark. The affinity of UPF1 for RNA was determined in the same setup. 40 µl of each binding reaction was transferred to a flat-bottom black 384-well plate (PerkinElmer OptiPlate 384-F) to measure the fluorescence polarization at 25 °C using a Spark multimode microplate reader (Tecan Lifesciences). The value obtained in absence of any protein (RNA alone) was considered as baseline and subtracted from all the other fluorescence polarization (F.P) values. Fluorescence anisotropy was calculated from fluorescence polarization using the formula [2*F.P/(3-F.P)] and normalized against the value obtained for the highest N-concentration. The data presented are an average of at least three independent sets of experiment and were fitted to an equation representing one site-specific binding with Hill slope in GraphPad Prism 5.0. The error bars represent the standard deviation of each data point from the mean, while the error associated with the K_D denotes standard error of mean (SEM).

Fluorescence-based unwinding assay

The RNA substrate (5'-GGGACACAAAACAAAAGACAAAAACACAAAACAAAAGACAAAAA CACAAAACAAAAGACAAAAAGCCAAAUUACCGUGUGCGUACAACUAGCU-3') used in this helicase assay were prepared by in vitro transcription (IVT) from a linearised dsDNA template (5'-CTAATACGACTCACTATAGGGACACAAAACAAAAGACAAAAACACAAAACAA AAGACAAAAACACAAAACAAAAGACAAAAAGCCAAATTACCGTGTGCGTACAACTAGCT-3') using the oligos oAA81 and oAA82 (Supplementary Table 2). Fresh helicase substrate (RNA:DNA duplex at a 11:7 ratio) was prepared prior to every experiment by incubating the Alexa Fluor 488-labeled DNA (5'-Alexa Fluor 488-AGCTAGTTGTACGCACAC-3') and transcribed RNA with 2 mM magnesium acetate and 1x unwinding buffer (10 mM MES pH 6.5, 50 mM potassium acetate, 0.1 mM EDTA) at 95 °C for 3 mins and 30 sec, followed by slow-cooling the mixture to 30 °C. For each replicate, a 40 µl reaction mixture was prepared which included 16.5 µl of reaction mixture and 2 µl of each protein of interest (final concentrations of 300 nM UPF1, 600 nM UPF2 and increasing concentrations of Nfl from 75 nM-1200 nM). The 3'-BHQ1-labeled quencher (5'-GTGTGCGTACAACTAGCT-BHQ1-3') was added to the mixture at a final concentration of 0.56 µM (4X of labeled DNA) as a trap.

The reaction mixture containing UPF1 or UPF1-UPF2 was incubated at 25 °C for 10 mins.

↓

Addition of N-protein + another 10 mins of incubation

↓

1.5 µl of 15 µM 3' BHQ1 quencher

The whole mixture of 24 µl was prepared in the dark in opaque reactions tubes and subsequently transferred into a black, flat-bottomed, 384-well plate (PerkinElmer OptiPlate 384-F). 16 µl of 5 mM ATP (final concentration of 2 mM) was injected using the injector module of the plate reader. The change in fluorescence was monitored for 30 min at 30 °C. The measured fluorescence intensities were normalized to the 0-time point (baseline) for each condition to obtain relative fluorescence.

**Composition of master mix**	**Volume**	**Final Conc.**
5x Unwinding buffer	8 µl	1x
100 mM magnesium acetate	0.8 µl	2 mM
100 mM DTT (freshly prepared)	0.8 µl	2 mM
Duplex (RNA-DNA)	5.5 µl	75 nM
Nuclease-free water	1.4 µl	

Supplementary Table 1. Plasmids used in this study

E. coli expression (Vector backbone for all plasmids: pET28)

Serial No.	Gene	Construct	Info	Expression tags
1	UPF1#	115-914	CH-helicase domains	N-term, His_6
2	$UPF1_{CH}$#	115-283	CH domain	N-term, His_6
3	UPF2#	121-1227	MIF4G1-2-3 + UPF1 binding site	N-term, His_6
4	UPF2#	121-761	MIF4G1-2	N-term, His_6
5	UPF2#	761-1227	MIF4G3 + UPF1 binding site	C-term, His_6
6	UPF2#	1015-1227	UPF1 binding site	N-term, His_6
7	UPF2#	761-1106	MIF4G3 + acidic linker	N-term, His_6
8	UPF2#	761-1227	MIF4G3 + UPF1 binding site	N-term, GST-His_6
9	UPF3#	41-143	RRM of UPF3	
10	$MOV10_{fus}$#	MOV10 (1-264) GSAGAAGSGA -UPF1(295-914)	Fusion of MOV10 N-terminus and the helicase core of UPF1	N-term, His_6-Trx
11	Nfl*	1-419	Full-length	
12	N_{NTD}	1-247	IDR1+ RNA binding domain (RBD) + IDR2	
13	N_{RBD}	47-180	RBD	N-term, His_6
14	N_L	180-247	IDR2 between RBD and Dimerization Domain	
15	N_{CTD}	180-419	IDR2 + DD + IDR3	
16	N_{DD}	247-371	Dimerization domain	
17	$N_{L\text{-}DD}$	180-371	Linker + DD	
18	$N_{\Delta L}$	1-419	Full length, IDR2 is replaced by 12 a.a.(GSASGSGAGAGS)	
19	$N_{IDR1\text{-}Core}$	1-371	IDR1+RBD+DD, IDR2 is replaced by 12 a.a. (GSASGSG AGAGS)	
20	N_{Core}	47-371	RBD+DD, IDR2 is replaced by 12 a.a. (GSASGSGAGAGS)	
21	$N_{IDR1\text{-}RBD}$	1-180	IDR1 + RBD	
22	Nfl	1-419	Full-length	N-term, GST-His_6
23	GST		Full-length**	N-term, His_6
24	UPF1#	115-914	CH-helicase domain	N-term, GST-His_6

Mammalian expression (Vector backbone for plasmids 1-5: PB-CuO-BGH)

1	mGold	1-239	Full length	FLAG
2	Nfl	1-419	Full-length	FLAG
3	UPF1-R843C	1-1118	Full-length; R843C mutant	FLAG
4	TRS-L-WT-Globin	Complex	75-nt TRS-L + WT-globin gene (exon1-3 including introns)	-
5	TRS-L-PTC39-Globin	Complex	75-nt TRS-L + PTC39-globin gene (exon1-3 including introns)	-
6	Nfl	1-419	Full-length; Vector: pCDNA3.1	HA

* Gift from Markus Wahl, Freie Universität Berlin; ** Gift from Elena Conti, Max Planck Institute of Biochemistry, Martinsried; #These plasmids were already available in the lab.

Supplementary Table 2. Primers used in this study

Primer name	Description	Sequences
oMM1F	NCP_1_N_3CLIC	CCAGGGGCCCGACTCGATGAGCGATAATGGGCCG
oMM2F	NCP_47_N_3CLIC	CCAGGGGCCCGACTCGATGAACAATACCGCTAGTTGG
oMM3F	NCP_180_N_3CLIC	CCAGGGGCCCGACTCGATGTCACAGGCGAGTTCACG
oMM4F	NCP_247_N_3CLIC	CCAGGGGCCCGACTCGATGACCAAGAAATCTGCCGC
oMM5R	NCP_247_C_3CLIC	CAGACCGCCACCGACTGCTTAGGTCACCGTTTGACCTTGC
oMM6R	NCP_180_C_3CLIC	CAGACCGCCACCGACTGCTTATGAGCCACCGCGGC
oMM7R	NCP_419_C_3CLIC	CAGACCGCCACCGACTGCTTACGCCTGGGTACTATCCGC
oMM8R	NCP_371_C_3CLIC	CAGACCGCCACCGACTGCTTAGTCTTTCTTCGGCTCTGTCG
oMM11F	RBD-ΔIDR2-DD(12 a.a GSASGSGAGAGS)	GTGGCTCAGGTTCTGCTAGCGGATCGGGCGCAGGGGCCGG TTCAACCAAGAAATCTGCC
oMM12R	DD-ΔIDR2-RBD (12 a.a. GSASGSGAGAGS)	CTTGGTTGAACCGGCCCCTGCGCCCGATCCGCTAGCAGAAC CTGAGCCACCGCGGCTCC
oAA81	IVT CL template rev	AGCTAGTTGTACGCACACggtaatttggCTTTTTGTCTTTTG TTTTGTGTTTTTGT
oAA82	IVT CL template fwd	CTAATACGACTCACTATAGGGACACAAAACAAAA
P889	globin_qPCR_se	AAGGCTCATGGCAAGAAAG
P890	globin_qPCR_as	ACACCAGCCACCACTTTC
P1676	SRSF2_e2_se	GAATCCAAATCCAGGTCGC
P1677	SRSF2_e3_as	CCAGTTGCTTGTTCCAAGGA
oMM30F	SARS CoV-2 TRS-L 5'-UTR	CTAGCattaaaggtttataccttcccaggtaacaaaccaacc aactttcgatctcttgtagatctgttctctaaacgaacC
oMM31R	SARS CoV-2 TRS-L 5'-UTR	TCGAGgttcgtttagagaacagatctacaagagatcgaaagt tggttggtttgttacctgggaaggtataaacctttaatG
